# Brown Adipose Tissue: Molecular Mechanisms of Regulation, Physiological Functions, and Therapeutic Targets

**DOI:** 10.1002/mco2.70976

**Published:** 2026-09-20

**Authors:** Xiaoli Deng, Yuanyuan Shen, Ying Sun, Lu Lu, Chongkui Sun

**Affiliations:** ^1^ Department of Geriatrics, Institute of Geriatrics Sichuan Provincial People's Hospital University of Electronic Science and Technology Chengdu China; ^2^ State Key Laboratory of Swine and Poultry Breeding Industry College of Animal Science and Technology Sichuan Agricultural University Chengdu China

**Keywords:** aging, batokines, brown adipose tissue, metabolic diseases, thermogenesis

## Abstract

Brown adipose tissue (BAT) biology has undergone a profound evolution over the past two decades, expanding from a narrow focus on thermogenic capacity in rodents to the establishment of its pleiotropic roles in human metabolic regulation. While this transition has enabled major progress in defining underlying molecular mechanisms and strengthened the clinical rationale for targeting BAT, it has also exposed unresolved challenges and knowledge gaps. In this review, we synthesize current evidence across the field, integrating findings from molecular, physiological, and translational studies to delineate the hierarchical regulatory networks that govern BAT development and function. We focus on three themes: first, the transcriptional, epigenetic, and posttranscriptional mechanisms that establish and maintain BAT identity; second, the expanding spectrum of BAT's physiological functions beyond thermogenesis, including its crosstalk with other metabolic organs and the immune system; and third, the emerging strategies and persistent obstacles in harnessing BAT's therapeutic potential for obesity, diabetes, and related metabolic disorders. By consolidating these perspectives, we propose an integrative framework for understanding BAT as a central hub in human metabolic regulation while identifying critical knowledge gaps that warrant further investigation.

## Graphical abstract

This review systematically covers three core dimensions of brown adipose tissue (BAT): multilayered regulatory mechanisms governing brown adipocyte differentiation and phenotypic identity, physiological functions and signaling pathways in mediating thermogenesis and systemic metabolic crosstalk between BAT and multiple organs, and emerging clinical in vivo diagnostic and potential therapeutic strategies targeting BAT for metabolic and aging‐related disorders.

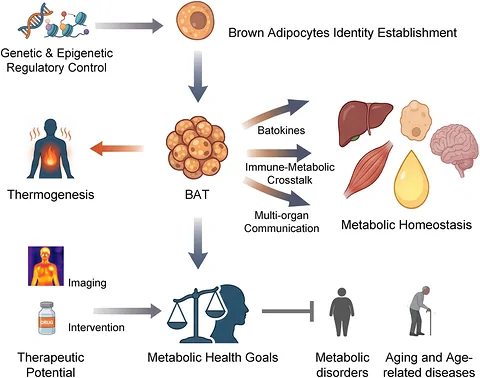

## 1 Introduction

In mammals, adipose tissue is not only an energy storage organ but also a key regulator of thermoregulation, energy metabolism, and endocrine signaling. Accounting for 4–40% of total body mass in adults [1], this dynamic organ is primarily classified into white adipose tissue (WAT) and brown adipose tissue (BAT) based on cellular structure and physiological function. White adipocytes are primarily responsible for energy storage and systemic metabolic regulation, whereas brown adipocytes specialize in thermogenesis. Beyond these two well‐established types, a third, inducible type of thermogenic adipocyte, termed beige adipocyte, has been identified, adding a layer of cellular plasticity and metabolic complexity to adipose biology [2, 3]. The thermogenic capacity of brown/beige adipocytes stems from uncoupling protein 1 (UCP1) [4]. Unlike in most cells, where the mitochondrial proton gradient drives ATP synthesis [5], UCP1 dissipates the gradient as heat instead of producing ATP [6]. This mechanism of uncoupled respiration makes BAT a key effector of nonshivering thermogenesis (NST) [7].

The discovery and characterization of BAT have a long history spanning centuries, yet its physiological relevance in humans has only recently been fully appreciated. BAT was first described in the 16th century by Swiss naturalist Conrad Gessner, who noted this tissue as “neither fat, nor flesh, but something in between” [8]. Its presence in human infants was confirmed in the early 20th century, where it was recognized as critical for maintaining body temperature during the neonatal period [9]. For much of the subsequent decades, BAT was considered to be a developmentally regressive tissue that gradually disappeared after infancy, and its study was largely confined to small mammals, particularly rodents, in which its thermogenic function was well established [10]. This view began to be challenged by Heaton's autopsy studies in the 1970s, which demonstrated that BAT depots could be identified in human adults, albeit at reduced levels compared with infants [11]. However, the lack of noninvasive detection methods limited further investigation until the landmark application of ^18^F‐fluorodeoxyglucose (^18^F‐FDG) positron emission tomography‐computed tomography (PET‐CT) in 2009, which unequivocally confirmed the presence of metabolically active BAT in adult humans and revealed its cold‐inducible and glucose‐avid nature [12, 13, 14, 15]. These seminal findings overturned the long‐standing dogma and ushered in a new era of BAT research, rapidly expanding the field from a narrowly focused thermogenic curiosity to a major area of metabolic investigation [16].

The concurrent global rise in the prevalence of obesity and related metabolic diseases, such as Type 2 diabetes, nonalcoholic fatty liver disease, and cardiovascular disease, underscores the urgent need for novel therapeutic strategies [17]. In this context, BAT activation represents a promising approach. In rodents, sustained BAT activation promotes energy dissipation, leading to negative energy balance and weight loss [18]. Crucially, this observation is relevant to humans: obese and diabetic individuals exhibit reduced BAT mass and activity, a deficiency that can be partially reversed by cold exposure [19, 20]. Cypess et al. reported an inverse correlation between BAT prevalence and body mass index (BMI), as well as fasting blood glucose levels, strongly suggesting that enhancing BAT thermogenic activity could combat obesity and associated metabolic disorders [15]. Mechanistically, BAT‐mediated energy expenditure can be augmented both by activating existing BAT and by inducing the “browning” of WAT to form beige adipocytes, thereby significantly increasing whole‐body thermogenic capacity [3]. These findings have positioned BAT as an attractive pharmacological target for metabolic disease intervention [21].

Beyond its direct thermogenic function, BAT exerts pleiotropic effects on systemic metabolism through multiple parallel mechanisms. First, BAT acts as a “metabolic sink,” actively clearing glucose, fatty acids, and branched‐chain amino acids (BCAAs) from the circulation, thereby improving whole‐body glucose homeostasis and lipid profiles [18, 19, 20, 21, 22]. Second, BAT functions as an endocrine organ, secreting a range of signaling molecules, collectively termed “batokines,” that communicate with distant organs, including the liver, skeletal muscle, and brain, to coordinate systemic energy balance, insulin sensitivity, and feeding behavior [23, 24]. For instance, BAT‐derived fibroblast growth factor 21 (FGF21) and neuregulin 4 (NRG4) have been implicated in hepatic lipid metabolism and adipose tissue remodeling, respectively [25]. Third, emerging evidence suggests that BAT plays a role in immunometabolism, modulating systemic inflammation through the clearance of BCAAs and the secretion of anti‐inflammatory factors [26]. However, BAT function declines progressively with age, characterized by mitochondrial dysfunction, attenuated sympathetic signaling, reduced thermogenic gene expression, and impaired adipocyte differentiation, a process that may exacerbate age‐related metabolic diseases [27, 28]. Understanding the mechanisms underlying this age‐associated decline has thus become an important research direction.

Thus, elucidating the biological characteristics of BAT and its mechanisms of action in physiology and disease has become a central focus of metabolic research. In this review, we systematically synthesize recent advances in the BAT field by focusing on three progressive and interconnected aspects. First, we delineate the molecular regulatory mechanisms governing BAT development and thermogenic function, including core transcriptional networks, epigenetic modifications, and posttranscriptional control. Second, building on this mechanistic foundation, we assess the broader physiological significance of BAT beyond thermogenesis, with particular emphasis on its systemic roles in glucose homeostasis, lipid clearance, and immunometabolism. Third, by integrating these mechanistic and functional insights, we evaluate the therapeutic potential of BAT, discussing clinical prospects and challenges associated with its activation via pharmacological, lifestyle, and advanced interventions for combating metabolic diseases. Through this structured exploration, we aim to provide an integrative framework for understanding BAT's multifaceted roles in human physiology and disease, while identifying critical knowledge gaps to guide future translational research.

## 2 Biological Characteristics and Molecular Regulation of BATs

### 2.1 Anatomical Distribution and Cellular Identity

Brown adipocytes and white adipocytes exhibit distinct morphological characteristics that reflect their divergent functions. Brown adipocytes are identified by numerous small lipid droplets and a centrally located nucleus [29]. They are enriched with large, abundant mitochondria featuring well‐defined lamellar cristae, which are essential for thermogenesis. In contrast, white adipocytes have a single large lipid droplet that displaces the nucleus toward the cell periphery. Their mitochondria are smaller, more elongated, and possess irregular cristae, reflecting their role in energy storage rather than energy dissipation [30].

WAT is the predominant form of adipose tissue in mammals, found in both subcutaneous and visceral depots. In females, subcutaneous WAT is particularly abundant in the breast and gluteal regions [31]. While the structure of human visceral adipose tissue resembles that of rodents, men lack epididymal adipose tissue.

The distribution of BAT is species specific and exhibits both evolutionary conservation and variation. In small rodents, BAT is primarily localized to the interscapular region. However, in humans, metabolically active BAT in adulthood is predominantly located in the cervical, supraclavicular, mediastinal, paravertebral, and periaortic regions [32]. This BAT is strategically distributed around major arteries, such as the aorta, carotid, and subclavian arteries, facilitating efficient heat transfer to the circulatory system through its pronounced vasculocentric distribution. Beige adipocytes, which are functionally similar to BAT, are primarily found in subcutaneous WAT depots in rodents and humans [33].

### 2.2 Developmental Lineage and Cellular Plasticity

The development of adipose tissue initiates during embryogenesis and continues through postnatal life, with distinct types of fat depots arising during specific developmental windows. White adipocytes have traditionally been considered mesoderm derived. However, recent studies indicate that some white adipocytes may also originate from neural crest cells of ectodermal lineage, broadening our understanding of their developmental origins [34]. Mature white adipocytes are derived from progenitor cells in WAT's stromal vascular fraction (SVF), a compartment that includes adipocyte precursors, stem cells, and immune populations like M2 macrophages and T cells [35, 36]. Various surface markers, such as PDGFRα and Sca1, have been identified on these progenitors, though the precise hierarchy remains under investigation [37].

Classical brown adipocytes arise from a distinct myogenic lineage during embryogenesis. They develop before WAT and play a critical role in NST, particularly at birth [38]. In mice, brown adipocytes derive from dermatome precursors that express somite markers, including *Pax7*, *En1*, *Myf5*, *Pax3*, and *Meox1* [39]. This lineage is conserved across species, with brown adipocytes in rodents and humans sharing a developmental origin with skeletal muscle. The master regulator PRDM16 functions as a key switch that drives these precursors toward a brown adipocyte fate while inhibiting myogenic differentiation [40]. Once committed to the brown adipocyte lineage, these cells maintain specific markers, such as *Lhx8*, *Zic1*, *Ebf2*, and *Prex1*, and exhibit an irreversible commitment to brown fat [41].

In contrast, beige adipocytes arise postnatally from a distinct lineage, more closely associated with the WAT lineage. They emerge in response to environmental stimuli such as cold exposure or β3‐adrenergic signaling. Beige progenitors express markers including *Pdgfrα*, *Sca1*, *Acta2*, and *Cd81* [42]. Cold‐induced beige adipogenesis is driven by the transcription factor EBF2, which acts as a specific marker for beige adipocyte differentiation in adult WAT [43]. Unlike brown adipocytes, which arise early in embryonic development, beige adipocytes can be induced throughout life under specific conditions [44]. Interestingly, beige adipocytes possess a remarkable degree of plasticity, as they can be generated either by the differentiation of dedicated precursor cells or by direct transdifferentiation of existing white adipocytes. The environmental conditions, such as temperature and β3‐adrenergic receptor activation, determine the route through which beige adipocytes are formed (Table 1 for a summary of different developmental origins, characteristics, and regulatory signals).

**TABLE 1:** Developmental origins of brown, beige, and white adipose tissue.

Type	Location	Developmental origins/precursor type
Brown 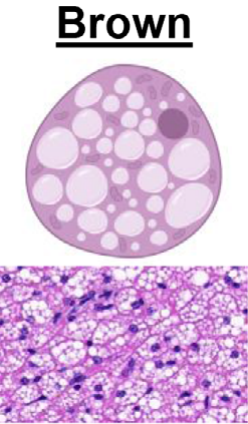	Interscapular Cervical Axillary Perirenal	Myf5+, Pax7+, En1+ cells in dermomyotome; derived from myogenic lineage, related to skeletal muscle development
Beige 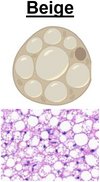	WAT depots: Inguina, epididymal	Ebf2+, PDGFRα+ cells, Acta2+ smooth muscle cells, Myh11+ smooth muscle cells, Pdgfrβ+ mural cells, bipotent PDGFRα+ precursor
White 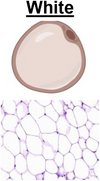	Subcutaneous Visceral	Wt1+ mesothelial cells (visceral); PDGFRα+, Sca1+ precursor cells in the stromal SVF

In humans, adipose tissue shows characteristics that closely resemble murine beige fat, although significant heterogeneity exists across depots and individuals. Transcriptomic profiling reveals high expression of beige‐selective markers, such as CD137, TMEM26, and TBX1, in human adipose tissue [45]. Notably, human supraclavicular BAT expresses classical brown fat markers such as UCP1, ZIC1, and LHX8, suggesting a closer alignment with classical BAT [46].

### 2.3 The Molecular Machinery of Adipocyte Fate and Function

**FIGURE 1:**
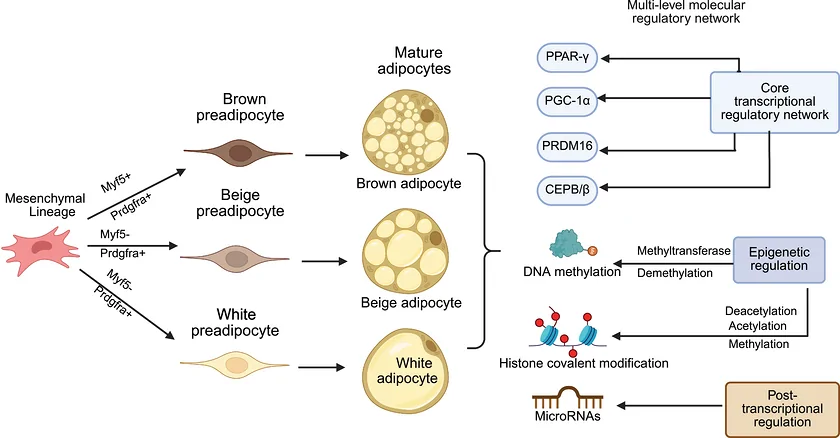
The hierarchical molecular network governing adipocyte fate and browning. Adipocyte browning is governed by a multilayered molecular network. Core transcription factors (PPARγ, C/EBPβ, PRDM16, PGC‐1α) drive thermogenic differentiation, while epigenetic modifications (histone/DNA remodeling) modulate chromatin accessibility. Concurrently, microRNAs fine‐tune key signaling pathways. This hierarchical system ultimately dictates the balance between white fat storage and brown/beige energy dissipation.

Adipogenesis, the process by which precursor adipocytes differentiate into mature lipid‐laden adipocytes, is tightly controlled by multilayered molecular networks [47, 48]. This section systematically delineates three core regulatory tiers governing the fate and function of brown and beige adipocytes: the core transcriptional network, epigenetic modifications, and posttranscriptional mechanisms (Figure 1).

#### 2.3.1 Core Transcriptional Regulatory Network

Research has identified approximately 50 transcriptional and epigenetic regulators that modulate brown and beige adipocyte development. Strikingly, most of these factors converge on four core regulators: PPARγ, C/EBPβ, PRDM16, and PPARγ coactivator‐1α (PGC‐1α). Among them, PPARγ and C/EBPβ function as essential transcription factors that initiate and maintain adipocyte differentiation, whereas PRDM16 and PGC‐1α act as key coregulators, with PRDM16 determining metabolic identity and PGC‐1α amplifying thermogenic signals. Collectively, these components form a tightly coordinated network that precisely controls the adipose browning program.

PPARγ serves as the master regulator of adipogenesis and is indispensable for both white and brown adipose lineages [49, 50]. Beyond driving differentiation, it functions as a central platform for integrating multiple signaling inputs. Its transcriptional activity is finely modulated by external stimuli and interacting coregulators. For instance, PRDM16 forms a potent activating complex with PPARγ, robustly driving the brown/beige gene program [51, 52]. In contrast, the corepressor TLE3 competitively binds PPARγ, antagonizing PRDM16 action and thereby suppressing browning [52]. Furthermore, the effects of numerous browning agents ultimately converge on the PPARγ pathway. Thiazolidinediones, for example, act as PPARγ agonists that promote browning [53], and the metabolic benefits of Gleevec have also been shown to depend on PPARγ‐mediated signaling.

C/EBPβ acts as an early‐response driver to external cues such as cold exposure. Its expression is significantly higher in BAT than in WAT and is potently induced by cold stimulation. The regulation of C/EBPβ occurs at multiple levels. Transcriptionally, its expression is activated by PLAC8 [54]. Posttranscriptionally, it is modulated by microRNAs (miRNAs) such as miR‐155, which directly targets its mRNA, and miR‐196a, which enhances its expression by inhibiting its transcriptional repressor HoxC8 [55, 56, 57].

PRDM16 serves as the central molecular switch determining brown and beige adipocyte identity [58]. Initially thought to bind DNA directly via its zinc finger domain [59], subsequent studies revealed that point mutations disrupting DNA binding do not impair its ability to promote BAT formation, confirming its primary role as a transcriptional coregulator. Its mechanism involves bidirectional regulation. On one hand, it interacts with DNA‐binding proteins such as PPARγ and C/EBPβ to recruit activating complexes to target genes, strongly activating BAT‐selective gene programs [60], on the other hand, it actively represses white adipocyte‐specific genes by forming inhibitory complexes with CtBP1/2, thereby refining lineage commitment [61]. In beige fat, PRDM16 expression is equally critical for activating beige‐selective gene programs [62], and its ablation severely compromises beige adipocyte development [63, 64].

PGC‐1α does not determine cell fate but functions as an environmental sensor and amplifier of the thermogenic response [65, 66]. Its expression is robustly induced by cold exposure or cAMP signaling. As a coactivator, it interacts with nuclear receptors such as PPARγ and thyroid hormone receptors, as well as transcription factors like IRF4, and is recruited to the regulatory regions of genes including *UCP1*, potently driving mitochondrial biogenesis and thermogenic gene expression [67, 68, 69]. Multiple hormones and factors, such as growth factor 21 (FGF21), promote adipose thermogenesis by inducing PGC‐1α, and its presence is required for FGF21‐induced thermogenic output [70]. Concurrently, its activity is under stringent negative regulation by several factors. RIP140, SRC2, and TWIST1 directly bind and inhibit PGC‐1α [71, 72, 73, 74], while Rb and p107 suppress its gene expression to limit thermogenesis [75, 76]. Although essential for thermogenic function, PGC‐1α deletion does not alter brown adipocyte fate or BAT mass, confirming its role as a signal amplifier rather than a fate determiner [77].

In summary, these four core factors constitute a hierarchical and functionally complementary regulatory network. PPARγ and C/EBPβ establish the transcriptional foundation for adipocyte differentiation. PRDM16 then acts as the decisive fate switch through bidirectional gene regulation, and PGC‐1α integrates and amplifies upstream physiological and pathological signals into efficient thermogenic responses.

#### 2.3.2 Epigenetic Regulation: Histone Modifications and DNA Methylation

The differentiation and function of brown and beige adipocytes are governed by a unique gene expression program that is precisely regulated at multiple levels, with epigenetic mechanisms playing a central role. Histone covalent modifications constitute one of the key regulatory mechanisms. Histone acetyltransferases activate thermogenic gene programs by catalyzing acetylation of histone lysine residues, thereby relaxing chromatin structure. Specifically, CBP/p300 is indispensable for brown and beige adipocyte differentiation and responds to cold signaling by catalyzing H3K27ac, a marker associated with active enhancers, and activating PPARγ [78, 79, 80, 81]. Similarly, GCN5/PCAF promotes this process through catalyzing H3K9ac and acetylating C/EBPβ [82, 83]. In contrast, histone deacetylases such as HDAC3 and HDAC11 suppress thermogenic programs by removing H3K27ac [84, 85]. Members of the Sirtuin family demonstrate complex functions: SIRT1, SIRT3, and SIRT6 play distinct yet crucial roles in adipose browning and mitochondrial function through deacetylation of specific substrates [86, 87, 88].

Regarding histone methylation, methyltransferases that catalyze activating marks, particularly MLL3 and MLL4, are essential for brown adipocyte differentiation through deposition of H3K4me1 and H3K4me2 [89, 90]. Interestingly, EHMT1, which catalyzes repressive marks, positively regulates thermogenesis via interaction with PRDM16 [91]. Demethylases including KDM3A and KDM6B serve as crucial drivers of thermogenic gene activation, such as *Ucp1* expression, by removing repressive H3K9me2 and H3K27me3 marks [92, 93, 94].

Beyond histone modifications, DNA methylation significantly influences adipocyte fate determination. Methylation patterns established and maintained by DNA methyltransferases generally suppress gene expression. Research demonstrates that the DNA methylation inhibitor 5‐azacytidine promotes adipogenesis, indicating DNA methylation's basal inhibitory role in adipogenic programming [95]. In thermogenic regulation, demethylation of the *UCP1* enhancer is critical for cold‐induced expression [96, 97]. Mechanistically, *Dnmt1* represses myogenic genes in BAT to ensure proper lineage commitment [98], while *Dnmt3a* deletion improves metabolic phenotypes by upregulating FGF21 [99]. Conversely, knockout of DNA demethylase *Tet1* increases energy expenditure and protects against obesity, though potentially through mechanisms independent of its canonical enzymatic activity, suggesting complex regulatory networks [100].

#### 2.3.3 Posttranscriptional Regulation: The Role of MicroRNAs

miRNAs are a class of single‐stranded, noncoding RNA molecules, 20–24 nucleotides in length, that finely regulate gene expression at the posttranscriptional level and are involved in cellular metabolism and the pathology of various diseases [101]. Recent studies have revealed that multiple miRNAs play key roles in regulating the differentiation and function of beige and brown adipocytes, offering new avenues for treating obesity and related metabolic diseases by increasing energy expenditure [102].

In the regulation of brown adipogenesis, several miRNAs have been identified that specifically regulate thermogenesis. For example, inhibition of miR‐182 and miR‐203 significantly reduces BAT markers such as UCP1, PGC1‐α, and Cidea, and downregulates genes involved in the electron transport chain [103]. Their targets have been identified as *Pdgfrα* and *Insig1*. The miR‐193b‐365 cluster promotes BAT differentiation by inhibiting *Runx1t1*, although its function in vivo has not yet been fully confirmed [104]. miR‐328 is upregulated during brown adipocyte differentiation. Its inhibition reduces the expression of thermogenic genes such as *Ucp1* and *Prdm16*, while its overexpression reduces energy expenditure by inhibiting Bace1 protein [105]. The miR‐106b‐93 cluster, a negative regulator of brown adipocyte differentiation, is upregulated in obese mouse models [106]. Its inhibition induces enhanced expression of markers such as UCP1 and PRDM16, suggesting a key role for this cluster in energy homeostasis [107]. Furthermore, elevated circulating levels of liver‐specific miR‐122 in obese individuals are negatively correlated with brown adipocyte activity [108].

In the field of beige adipocyte regulation, several key miRNAs have been shown to be involved in developmental and functional regulation. miR‐196a induces white adipocyte browning by directly targeting *Hoxc8* which is a repressor of C/EBPβ [109]. Its overexpression improves glucose metabolism and enhances obesity resistance. The miR‐26 family is induced by cold exposure and promotes the expression of genes such as UCP1 and PGC‐1α by targeting ADAM17, accelerating the process of adipose browning. In contrast, miR‐125‐5p, a negative regulator of browning, is highly expressed in white fat. Its injection significantly inhibits beige adipogenesis and mitochondrial biogenesis [110].

Notably, some miRNAs regulate both brown and beige fat development. The miR‐30 family is highly expressed in BAT and is induced by β‐AR signaling or cold exposure. It positively regulates thermogenic gene expression and mitochondrial respiration by targeting *RIP140* [111]. miR‐455 is induced by cold exposure and BMP7, and transgenic mice overexpressing it exhibit significant white fat browning and improved metabolism [112]. miR‐378 exhibits dual regulatory capabilities in that it promotes brown adipogenesis by targeting *Pde1b* while also inhibiting white fat browning. This unique property occupies a unique position in metabolic regulation [113].

These miRNAs collectively maintain or challenge cellular identity by finely regulating gene expression networks, forming a complex regulatory network. Although a large number of miRNAs have been identified as involved in fat metabolism regulation, their specific mechanisms of action remain largely undefined. Further in‐depth research on the regulatory networks of these miRNAs is needed to provide a theoretical basis for the development of miRNA‐based obesity treatment strategies.

## 3 Neural and Metabolic Signal Transduction Pathways in Adipose Thermogenesis

### 3.1 The Neural Circuitry of Thermoregulation

**FIGURE 2:**
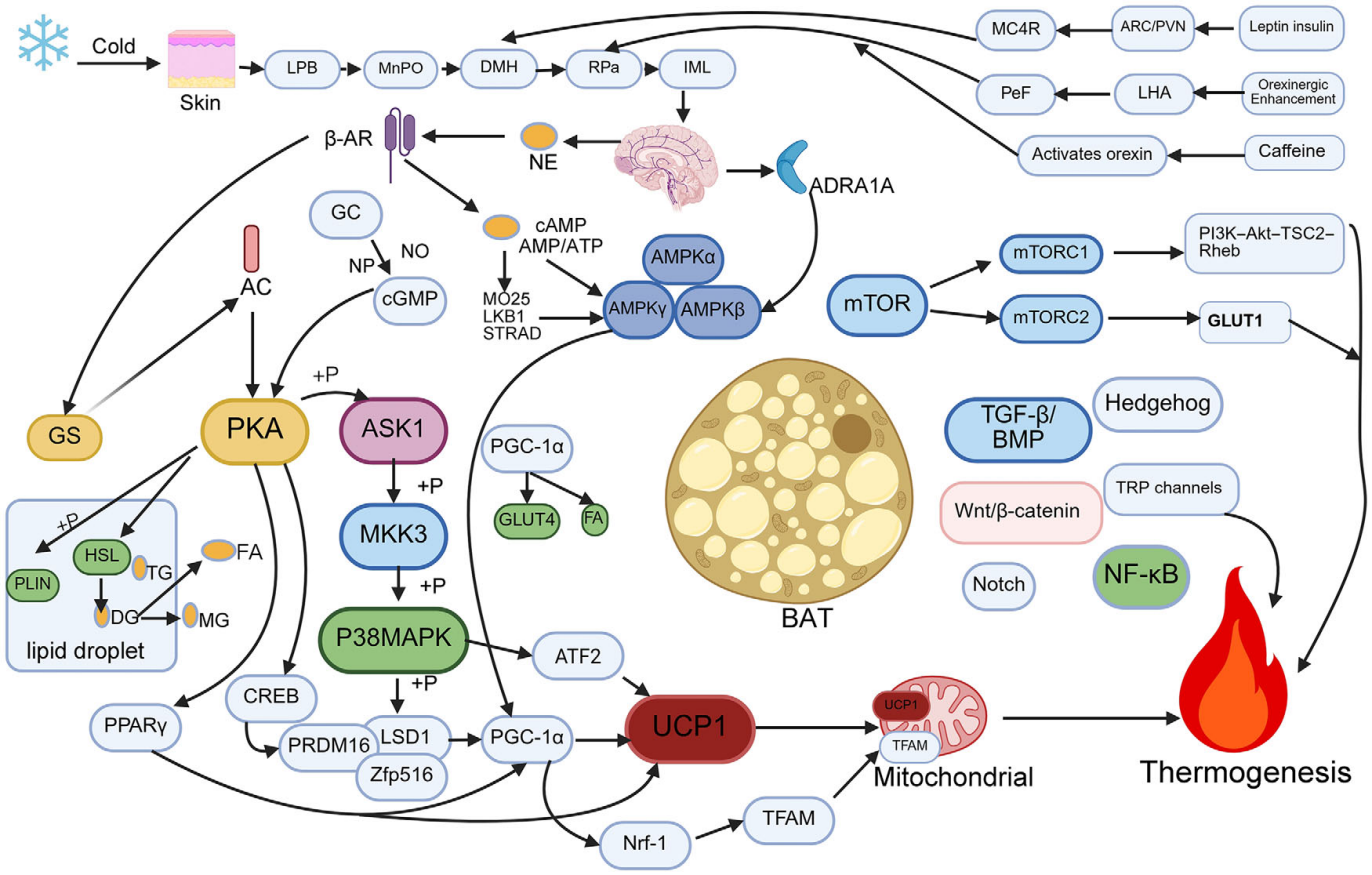
Central and peripheral signaling pathways regulating thermogenesis in brown adipose tissue. Cold signals activate the hypothalamic–sympathetic neuroaxis, triggering norepinephrine release in BAT. β‐Adrenergic stimulation drives the cAMP–PKA cascade, inducing lipolysis and UCP1‐mediated thermogenesis. This core pathway is modulated by cGMP/PKG, AMPK, mTOR, and BMP/Smad signaling, while TRP channels and Wnt/Notch pathways regulate progenitor fate. This integrated system dynamically coordinates BAT thermogenesis in response to environmental cues.

BAT function is exquisitely regulated by the sympathetic nervous system (SNS), which is governed by a multilayered neural network within the central nervous system (CNS). Sympathetic output is central to the homeostatic balance between energy expenditure and caloric intake. In adipose tissue, norepinephrine (NE) released from sympathetic nerve terminals serves as the primary signal for activating lipolysis in WAT and thermogenesis in BAT [114] (Figure 2). While the neural circuitry underlying BAT thermoregulation is relatively well characterized in rodents [103], the central control of human BAT remains incompletely understood, relying largely on indirect evidence such as adrenergic receptor expression in BAT [115] and thermogenic responses to cold or adrenergic agonists [116, 117, 118].

Sympathetic control of BAT involves a hierarchical network extending from the cortex to the spinal cord, integrating inputs from two primary systems: thermoregulation and energy homeostasis. Human neuroimaging studies indicate that thermoregulation engages the cerebral cortex and hypothalamus [119]. Furthermore, human medullary raphe neurons are activated by thermal challenges, and their anatomical location corresponds to the rodent nucleus raphe pallidus (RPa), which is a key region housing BAT sympathetic premotor neuron [120]. The preoptic area acts as a central node for cold sensing. Although the medial preoptic area was traditionally viewed as suppressive, recent evidence has identified a critical upstream driver of BAT thermogenesis: an excitatory neuronal population within the median preoptic nucleus (MnPO) that expresses pyroglutamylated RF‐amide peptide (QRFP) [121, 122]. These MnPO neurons project directly to the dorsomedial hypothalamic nucleus (DMH), which is essential for cold‐induced thermogenesis [123]. The DMH serves as a pivotal relay, receiving glutamatergic input from the MnPO and transmitting signals downstream to the RPa [124].

Concurrently, energy status modulates BAT activity through the hypothalamic melanocortin system. MC4R neurons in the arcuate nucleus (ARC) and paraventricular nucleus (PVN) integrate metabolic signals from hormones such as leptin and insulin, thereby influencing thermogenic pathways and regulating energy expenditure. The RPa, located in the brainstem, is regarded as the final common pathway for BAT sympathetic output [125]. It integrates commands from upstream nuclei like the DMH and projects directly to sympathetic preganglionic neurons in the spinal cord's intermediolateral column (IML), ultimately stimulating BAT via NE release. In summary, within the canonical thermoregulatory pathway, a cold signal is first detected by cutaneous receptors. The signal is then relayed centrally to the lateral parabrachial nucleus and on to the MnPO. After integration in the DMH, the command is transmitted through the RPa and the spinal cord IML, eventually activating the sympathetic postganglionic neurons that trigger thermogenesis in BAT.

Multiple neuromodulators fine‐tune BAT activity within this complex circuitry. Orexin (hypocretin), produced by neurons in the lateral hypothalamic area and perifornical area, enhances BAT sympathetic nerve activity through projections to the RPa. Notably, low‐dose caffeine has been shown to promote thermogenesis without inducing anxiety, likely via activation of these orexinergic neurons [126]. Importantly, such stimulation selectively increases BAT thermogenesis in rats without elevating heart rate or mean arterial pressure, underscoring the feasibility of activating BAT‐specific pathways while avoiding systemic cardiovascular effects. In addition, QRFP‐expressing neurons in the MnPO have been identified as a key excitatory population driving thermogenesis. QRFP signals through its receptor GPR103. Intriguingly, this system not only influences appetite but also modulates thermogenesis, body weight, and lipogenesis, suggesting that targeting QRFP signaling may offer a novel approach to regulating energy balance.

Centrally derived signals ultimately act on BAT via NE binding to adrenergic receptors, a process marked by significant species differences. Rodent BAT function depends primarily on β3‐adrenergic receptors, whereas human BAT is mainly regulated by β1 and β2 subtypes. This distinction is supported by genetic studies showing that mice lacking β3‐adrenergic receptors maintain thermogenic capacity by upregulating UCP1 and β1 receptors. This compensatory mechanism may more closely resemble human physiology. Receptor activation initiates the cyclic adenosine monophosphate–protein kinase A (cAMP–PKA) signaling cascade, which stimulates lipolysis to supply free fatty acids (FFAs) as thermogenic substrates and activates downstream pathways such as p38 mitogen‐activated protein kinase (MAPK), ultimately leading to UCP1 expression and the conversion of chemical energy into heat.

Despite substantial advances in rodent models, translating these findings to humans remains challenging. Evidence from studies of rats suggests that neural pathways controlling BAT are largely distinct from those regulating the cardiovascular system and cutaneous vasomotion, providing a rationale for selective therapeutic activation.

### 3.2 Metabolic Signaling Pathways and Stress Sensors in Thermogenic Adipocytes

Following sympathetic activation, intracellular signaling pathways translate NE stimulation into precise metabolic responses. These cascades form an integrated network that regulates thermogenic programming through complementary mechanisms.

#### 3.2.1 cAMP–PKA Signaling Pathway

The cAMP–PKA axis represents the principal signaling pathway mediating BAT thermogenesis [127]. Upon binding to β‐adrenergic receptors, NE activates G‐protein‐coupled adenylyl cyclase, catalyzing cAMP production and subsequent PKA activation [128]. Activated PKA phosphorylates multiple downstream targets, including p38 MAPK and its transcription factors ATF‐2 and PGC‐1α, collectively enhancing UCP1 expression [129]. Concurrently, PKA stimulates hormone‐sensitive lipase (HSL) and adipose triglyceride (TG) lipase, promoting lipolysis and FFA liberation. Phosphorylation of perilipin removes its protective barrier from lipid droplets, facilitating efficient lipid mobilization [130].

#### 3.2.2 cGMP–AKT Signaling Pathway

Complementing the cAMP system, cyclic guanosine monophosphate (cGMP) serves as a key second messenger in thermogenic regulation [131]. Produced by guanylate cyclase in response to nitric oxide (NO) or natriuretic peptides (NPs), cGMP activates the PKG/AKT signaling cascade to promote lipolysis and UCP1 expression [132, 133, 134, 135, 136]. The NO–cGMP pathway ameliorates obesity through enhanced mitochondrial biogenesis and energy expenditure [137]. Similarly, the NP system, such as ANP, BNP, CNP, induces cGMP production via NPRA/NPRB receptors, contributing to both WAT browning and BAT thermogenesis [138].

#### 3.2.3 AMPK Signaling Pathway

AMP‐activated protein kinase (AMPK) functions as a central energy sensor coordinating metabolic adaptation in BAT. Highly expressed in thermogenic adipocytes, AMPK activation, which is typically triggered by an elevated AMP/ATP ratio, promotes glucose and fatty acid uptake, enhances mitochondrial β‐oxidation, and inhibits lipid and cholesterol synthesis, thereby increasing net energy expenditure [139]. Beyond acute metabolic regulation, AMPK is essential for maintaining BAT progenitor cell density and differentiation capacity, while its ablation leads to thermogenic impairment and obesity [140]. Notably, NE can increase PGC‐1α expression through an AMPKβ1‐dependent mechanism that mimics canonical AMPK signaling [141, 142, 143]. Molecular studies reveal that capsaicin activates an AMPK–SIRT3 positive feedback loop, epigenetically suppressing mitochondrial calcium uniporter‐mediated calcium overload and protecting BAT against “whitening” [144]. Importantly, AMPK also serves critical metabolic sensing functions in the CNS, particularly the hypothalamus. Hormones including leptin, thyroid hormone, and glucagon‐like peptide‐1 (GLP‐1) indirectly promote thermogenesis by inhibiting hypothalamic AMPK activity, thereby disinhibiting sympathetic outflow to BAT [145, 146].

#### 3.2.4 mTOR Signaling Pathway

The mechanistic target of rapamycin (mTOR) coordinates BAT energy metabolism and lipid handling through its distinct complexes, mTORC1 and mTORC2 [147]. mTORC1 exhibits differential effects on WAT versus BAT and integrates inputs from growth factors, nutrient availability, and multiple signaling pathways including Wnt, Hippo, and Notch [148]. Downstream, mTORC1 regulates substrates such as 4E‐BP, ULK1, and S6K through the PI3K–Akt–TSC2–Rheb axis, modulating thermogenic gene expression and autophagy. Meanwhile, mTORC2 participates in glucose homeostasis and lipid oxidation, enhancing glucose uptake via GLUT1 and regulating lipid breakdown through FoxO1‐mediated transcription [149].

#### 3.2.5 TGF‐β/BMP Signaling Pathway

The transforming growth factor‐β (TGF‐β) and bone morphogenetic protein (BMP) signaling pathway extensively regulates adipocyte proliferation, differentiation, and metabolic function. *Smad3* suppresses BAT differentiation and thermogenesis by binding and inhibiting *PGC‐1α* transcription [150]. BMP family members including BMP4, BMP7, and BMP8a/b exhibit sexually dimorphic and tissue‐specific effects. For instance, BMP8a induces thermogenesis exclusively in female mice [151, 152]. Central administration of BMP9 suppresses hepatic gluconeogenesis, whereas the BMP antagonist Noggin promotes WAT browning [153].

#### 3.2.6 Other Signaling Pathways

Transient receptor potential (TRP) channels influence BAT and WAT metabolism by modulating Ca^2+^ influx [154]. Thermo‐sensitive members demonstrate divergent effects: TRPV1/3 activation promotes thermogenesis, whereas TRPV2/4 inhibits it, and TRPP3 enhances mitochondrial function [155, 156].

Developmental and inflammatory pathways contribute significantly to thermogenic regulation. The Wnt/β‐catenin pathway typically suppresses adipogenesis and thermogenesis by inhibiting master regulators PPARγ and PGC‐1α [157]. Notch signaling inhibits browning in WAT, but may promote thermogenesis in BAT, suggesting context‐dependent functions [158]. Hedgehog (Hh) signaling suppresses browning during early adipogenesis, while its activation in specific microenvironments such as bone indirectly promotes WAT browning [159, 160]. The NF‐κB pathway, commonly associated with inflammation, has also been reported to indirectly modulate thermogenesis, as its downstream target IEX‐1 inhibits WAT browning, while Hoxa5 promotes this process by suppressing TLR4/NF‐κB signaling [161, 162].

## 4 The Bioenergetic and Endocrine Functions of BAT

### 4.1 Thermogenic Mechanisms

#### 4.1.1 UCP1‐Dependent Classic Mechanisms

**FIGURE 3:**
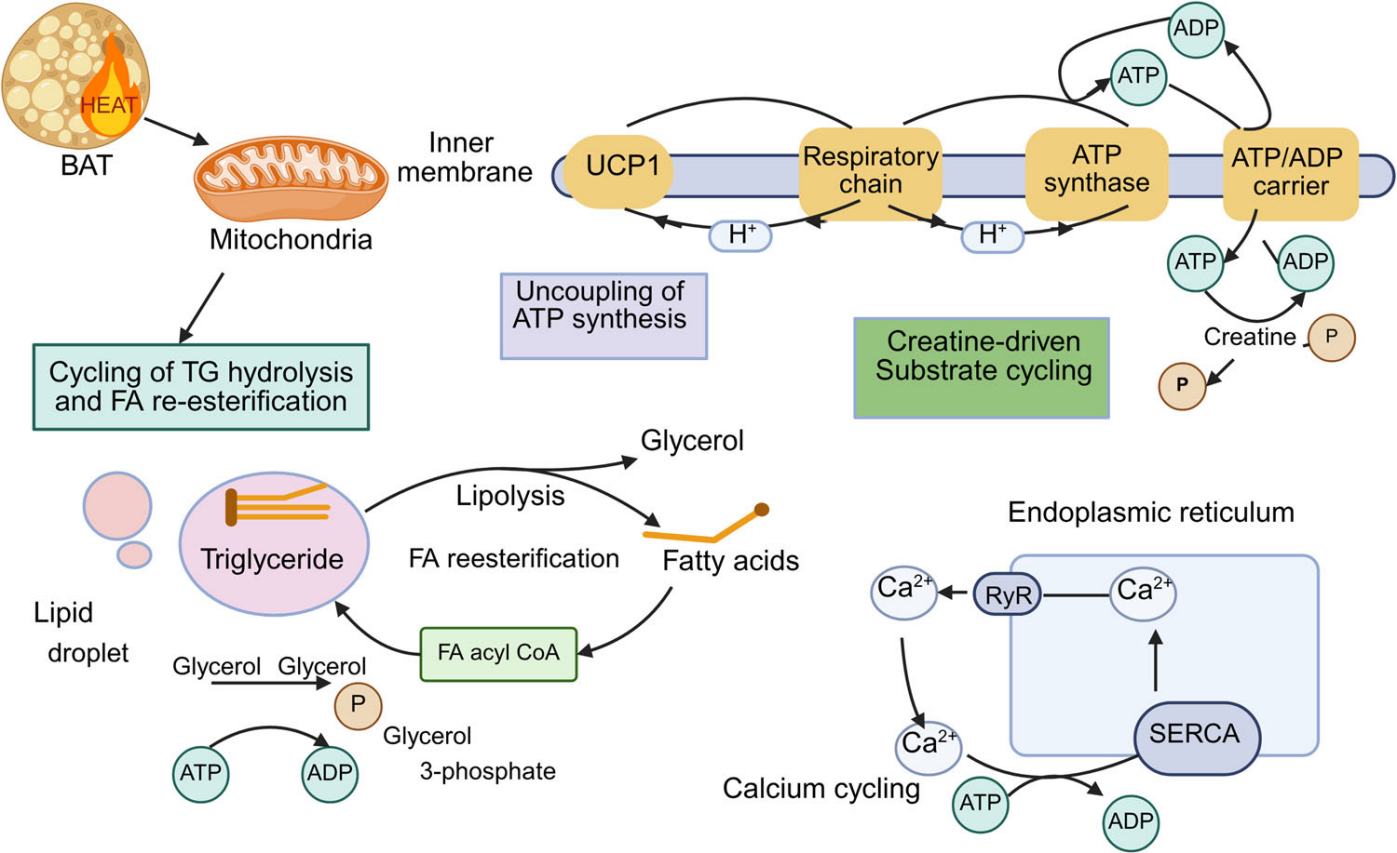
Canonical UCP1‐dependent and alternative UCP1‐independent thermogenic mechanisms in brown adipocytes. Brown adipocytes generate heat via canonical UCP1‐dependent proton leakage, which uncouples oxidative phosphorylation. This is complemented by three ATP‐consuming futile cycles: creatine cycling, Ca^2^^+^ cycling via RyR/SERCA, and triglyceride–fatty acid cycling. These UCP1‐independent mechanisms provide alternative thermogenic routes, particularly relevant when UCP1 activity is limited or in beige adipocytes.

According to Peter Mitchell's chemiosmotic theory, mitochondria typically establish a proton motive force through the respiratory chain to drive ATP synthesis. However, the mitochondria in BAT are a specialized exception. They are uniquely adapted for heat generation via uncoupled oxidative phosphorylation, with UCP1 serving as the essential mediator of this thermogenic process (Figure 3). UCP1, a 32 kDa member of the mitochondrial carrier family, is predominantly expressed in brown and beige adipocytes, where it executes a unique thermogenic role [163]. Physiological and genetic evidence firmly establishes its centrality that UCP1 is the principal driver of the remarkable thermogenic capacity in both rodent and human BAT. UCP1‐knockout mice develop severe hypothermia upon acute cold exposure, a phenotype not replicated by deletion of homologous proteins like UCP2 or UCP3, underscoring its indispensable role in adaptive thermogenesis [164, 165, 166].

Functionally, UCP1 acts as a regulated proton channel embedded in the mitochondrial inner membrane. By dissipating the proton gradient, it uncouples electron transport from ATP synthesis, thereby converting the energy derived from substrate oxidation directly into heat and alleviating respiratory inhibition caused by a high ATP/ADP ratio [167]. The activity of UCP1 is precisely regulated by a pair of molecular switches that function in opposition. Long‐chain fatty acids with a length of at least eight carbon atoms act as potent activators by directly binding to the protein and stimulating its proton conductance [168]. In contrast, purine nucleotides such as ATP, ADP, GTP, and GDP exert an inhibitory effect by binding to their specific sites, thereby effectively restraining UCP1's function [169].

Although the precise molecular mechanism continues to be refined, with models including allosteric regulation, cofactor requirements, or proton shuttles, the prevailing framework is the fatty acid cycle hypothesis. This model posits that UCP1 does not directly transport protons but instead acts as a fatty acid anion (FA^−^) transporter [170]. Driven by the membrane potential, UCP1 translocates FA^−^ into the intermembrane space, where protonation forms neutral fatty acids (FAH) that diffuse back into the matrix due to their hydrophobicity, completing a cycle with net proton influx [171]. Structural studies support this model by identifying specific basic residues within the UCP1 transmembrane domain that bind fatty acids [172]. This mechanism efficiently interrupts the two‐dimensional proton diffusion pathway from the pumping site to ATP synthase, achieving rapid uncoupling. Notably, UCP1 activation requires a specific cellular milieu, including not only high UCP1 expression but also reduced ATP synthase levels, metabolic pathways generating reducing equivalents, and an intact BAT biosynthetic system. Overexpression of UCP1 in nonthermogenic tissues like the heart fails to achieve effective uncoupling due to persistent purine nucleotide inhibition [173].

Quantification of UCP1 activity relies on proton leak kinetics as the gold standard. The core of this method lies in its simultaneous measurement of oxygen consumption rate and membrane potential. By using a TPMP^+^‐sensitive electrode to map leak respiration against a range of membrane potentials, it generates a functional profile that normalizes proton conductance to a common proton motive force. This normalization is crucial for making meaningful comparisons between different mitochondrial preparations or engineered UCP1 variants [174, 175]. This sensitive approach resolves subtle functional differences and has been successfully applied to compare UCP1 activity across species, proving powerful for quantifying evolutionary adaptation of UCP1 variants [176, 177].

While UCP1 is the predominant and physiologically well‐established thermogenic protein in mammals, other mechanisms contribute to heat production [178]. The basal proton leak, which is determined by the intrinsic permeability of the mitochondrial inner membrane, provides a background level of uncoupling. Other UCPs such as UCP2 and UCP3, though capable of uncoupling when ectopically overexpressed, exhibit far inferior efficiency due to their extremely low abundance in native tissues and are dispensable for thermoregulatory thermogenesis [179]. Thus, the mammalian thermogenic system displays a sophisticated hierarchy in that UCP1‐mediated uncoupling via the fatty acid cycle serves as the primary direct pathway, while basal proton leak and other UCP‐mediated processes provide supplementary routes.

#### 4.1.2 UCP1‐Independent Alternative Mechanisms

Although UCP1 is the core driver of adaptive thermogenesis, substantial evidence reveals multiple UCP1‐independent thermogenic pathways that consume ATP through futile metabolic cycles, collectively contributing to whole‐body energy homeostasis [180]. These mechanisms become particularly important during gradual cold adaptation in UCP1‐knockout mice, which can maintain body temperature through these compensatory pathways.

##### 4.1.2.1 Calcium Cycle Thermogenesis

This mechanism centers on the sarcoplasmic/endoplasmic reticulum calcium ATPase (SERCA). Under conditions of ADP limitation such as high ATP/ADP ratio and elevated cytosolic calcium (Ca^2+^), SERCA's ATP hydrolysis and Ca^2+^ pumping into the endoplasmic reticulum become uncoupled, resulting in continuous ATP consumption and heat release [181]. This mechanism has been studied in skeletal muscle. For example, myocardial isomerase (Sln) can uncouple its activity by binding to SERCA1 and promote thermogenesis [182]. Similarly, in beige adipocytes, the SERCA2B isoform expressed in them has also been shown to be an important non‐UCP1 thermogenic pathway [183]. This pathway is activated through a coordinated adrenergic response to cold. It is initiated both by α1‐adrenergic receptor stimulation, which mediates extracellular calcium influx, and by β3‐adrenergic receptor activation, which promotes calcium release from the endoplasmic reticulum via increased RyR2 activity [184]. Recent studies have also demonstrated that selective activation of the Ca^2+^ cycle in adipocytes by optogenetics is sufficient to trigger a significant thermogenic response and prevent diet‐induced obesity [184]. In addition, a low‐protein/high‐carbohydrate diet can also stimulate the SERCA pathway in WAT by activating AMPK, promoting thermogenesis [185, 186].

##### 4.1.2.2 Creatine Cycle Thermogenesis

This ineffective cycle involves the phosphorylation and dephosphorylation of creatine. Under ADP‐limited conditions, mitochondrial creatine kinase (Mi‐CK) catalyzes the phosphorylation of creatine to produce phosphocreatine, while phosphocreatine in the cytoplasm is hydrolyzed spontaneously or enzymatically, forming an ineffective substrate cycle, continuously consuming ATP and generating heat [187]. Genetic evidence shows that the loss of the rate‐limiting enzyme for creatine biosynthesis in adipose tissue or the cell surface creatine transporter (SLC6A8) reduces the overall energy expenditure of mice and leads to increased susceptibility to obesity, even in the presence of UCP1 [188, 189, 190, 191]. This proves that the creatine cycle is an important thermogenic pathway independent of UCP1, but how it is precisely induced and regulated in response to thermogenic stimulation remains to be further explored.

##### 4.1.2.3 TG‐Fatty Acid Cycle

This cycle involves the hydrolysis of TAG and the re‐esterification of fatty acids. Under the stimulation of fat browning signals, such as thiazolidinediones or cold exposure, lipolysis and fatty acid re‐esterification in adipose tissue are simultaneously activated, forming a futile cycle that consumes ATP [192, 193, 194]. It is estimated that about 14% of the energy expenditure stimulated by leptin treatment comes from the activation of this cycle [195]. Functioning in close relation to this cycle, the glycerol‐3‐phosphate (G3P) shuttle utilizes cycle‐derived G3P to transport cytosolic reducing equivalents into the mitochondria. Its unique property lies in the action of mitochondrial G3P dehydrogenase (mGPD), which feeds electrons directly into Complex III. This bypass of Complex I results in a lower ATP yield per oxygen atom consumed, thereby creating a less efficient energy conversion that inherently favors heat generation [196]. Supporting this view, mice with systemic mGPD knockout show reduced energy expenditure [197]. Interestingly, however, when UCP1 and mGPD are knocked out simultaneously, the energy expenditure of mice is actually higher than that of controls, accompanied by enhanced beige adipogenesis [198]. This suggests that when the main thermogenic pathway is blocked, the body can activate powerful compensatory mechanisms, which may involve the Ca^2+^ cycle or the creatine cycle, or there may be other pathways that have not yet been elucidated.

##### 4.1.2.4 Other Uncoupling and Regulatory Mechanisms

Mitochondrial uncoupling can also occur through UCP1‐independent pathways. A notable example involves the mitochondrial ATP/ADP translocase, which can mediate proton influx when stimulated by specific N‐acyl amino acids, such as C18:1‐Phe produced by the PM20D1 enzyme, thereby producing a significant uncoupling effect [199, 200]. In addition, thyroid hormone (TH) and its receptor also play an important role in UCP1‐independent thermogenesis. TH can significantly stimulate G3P shuttle activity in skeletal muscle and BAT. In mGPD knockout mice, a compensatory increase in UCP3 expression was observed, suggesting the existence of a UCP3‐dependent compensatory mechanism. It is worth noting that body temperature regulation not only depends on heat production but also involves the regulation of heat dissipation. Studies have found that mice carrying specific TRα1 mutations, despite increased BAT heat production, have increased heat dissipation due to vasoconstriction dysfunction, ultimately resulting in a decrease in body temperature. This reveals the complex mechanism by which the thyroid hormone system finely regulates body temperature balance beyond the scope of UCP1 [201, 202].

### 4.2 BAT as a Metabolic Sink

Activated BAT functions as a potent metabolic sink, demonstrating remarkable flexibility in substrate utilization for thermogenesis, which underpins its potential for improving systemic metabolic health.

#### 4.2.1 Glucose Metabolism

Human BAT exhibits substantial glucose uptake, a principle leveraged by ^18^F‐FDG‐PET imaging. Cold exposure increases this uptake by ∼50–200 nmol/g/min in healthy individuals [203, 204, 205]. While BAT's mass limits its contribution to whole‐body glucose disposal (<1%), its uptake is functionally critical. Inhibition of glycolysis or the glucose transporter GLUT4 impairs BAT thermogenesis [206, 207]. A major fate of glucose in human BAT is conversion to lactate, which is released; this may regulate local lipolysis via the GPR81 receptor and maintain redox balance [208, 209, 210, 211, 212]. The remainder of pyruvate enters mitochondria to fuel the TCA cycle and de novo lipogenesis (DNL), replenishing lipid stores [213]. Notably, reduced BAT glucose uptake in older or diabetic individuals does not always equate to impaired thermogenic capacity, as fatty acid oxidation may remain intact, challenging the sole reliance on ^18^F‐FDG‐PET for assessing BAT activity in metabolic disease [214].

#### 4.2.2 Lipid Metabolism

Lipids are the primary thermogenic fuel. Cold‐induced activation triggers intracellular TG hydrolysis, depleting lipid droplets—a conserved mechanism from rodents to humans, as confirmed by CT/magnetic resonance imaging (MRI) and microdialysis [215]. Pharmacological inhibition of this lipolysis ablates BAT thermogenesis [216]. To sustain energy output, BAT replenishes its TG pools by uptake of circulating FFAs and fatty acids from TG‐rich lipoproteins (TRLs) via LPL [217]. Chronic BAT activation with mirabegron improves blood lipid profiles, such as increased HDL‐C, likely through enhanced TRL clearance [218]. Glycerol kinase is highly expressed, enabling glycerol recycling for TG re‐synthesis and creating a substrate cycle that contributes to energy dissipation [219].

### 4.3 BAT as a Signaling Hub: Systemic Crosstalk in Metabolic Homeostasis

#### 4.3.1 The Batokines: Molecular Messengers of BAT

BAT synthesizes and releases a diverse array of peptides and metabolites, collectively termed “batokines,” which mediate biological effects via autocrine, paracrine, and potential endocrine pathways [220]. Proteomic analyses reveal the complexity of this secretory output; for instance, the human BAT secretome comprises at least 471 proteins, 101 of which are absent from the secretome of subcutaneous abdominal WAT. Through these secreted factors including encompassing proteins, lipids, and metabolites, BAT establishes an intricate intercellular communication network that plays a pivotal role in the fine‐tuning of systemic energy metabolism [221].

Among the numerous batokines, neuroregulatory protein 4 (Nrg4) is distinguished by its specific enrichment in BAT as an epidermal growth factor‐like ligand [222]. Although not essential for BAT development or acute cold‐induced thermogenesis, Nrg4 functions as a significant endocrine messenger [223]. It acts by activating the hepatic ErbB3/ErbB4 receptor–STAT5 signaling pathway, thereby effectively suppressing DNL mediated by the liver X receptor/sterol regulatory element‐binding protein 1c [224]. The physiological relevance of this axis is substantiated by studies in genetically modified models. Nrg4 deficiency exacerbates diet‐induced obesity and metabolic dysregulation, whereas its adipose tissue‐specific overexpression confers substantial protective effects [225].

Another key batokine, FGF21, exhibits its highest basal expression in the liver. However, under metabolic challenges such as cold exposure or BAT transplantation, BAT serves as a significant supplementary source of circulating FGF21 [226]. BAT‐derived FGF21 operates through multitiered regulatory mechanisms that it directly stimulates thermogenic gene programs in brown adipocytes and promotes the browning of WAT, while also enhancing sympathetic outflow via CNS actions, thereby establishing a positive feedback loop that amplifies thermogenesis [227].

From a structural and functional perspective, BAT‐secreted members of the vascular endothelial growth factor (VEGF) and BMP families are crucial. VEGF, a principal regulator of angiogenesis, promotes the proliferation of both endothelial cells and adipocyte progenitors via paracrine signaling, thereby supporting the development and maintenance of the dense vascular network essential for the high metabolic rate of BAT [228]. Concurrently, factors including BMP4, BMP7, and BMP8b act synergistically to regulate the commitment and differentiation of brown and beige adipocyte lineages [229]. BMP8b additionally exerts central regulatory functions by modulating hypothalamic signaling, which enhances sympathetic tone and systemic energy expenditure.

Within the local microenvironment, BAT‐derived interleukin‐6 (IL‐6) and nerve growth factor (NGF) contribute to immune and neural regulation, respectively. IL‐6 acts as a critical mediator improving glucose homeostasis following BAT transplantation, suppressing hepatic gluconeogenesis through activation of the STAT3 signaling pathway, thus illustrating a significant metabolic crosstalk between BAT and the liver [230]. NGF supports the establishment and plasticity of BAT sympathetic innervation by promoting the survival and axonal growth of sympathetic neurons [231]. Its expression is downregulated upon cold exposure, suggesting a primary role in developmental processes and homeostatic maintenance rather than in acute thermogenic activation [232].

It is important to note that the contribution of BAT to the circulating levels of classical adipokines is context‐dependent. For example, although BAT expresses adiponectin, its relative contribution to the systemic adiponectin pool may be minimal compared with other depots, such as bone marrow adipose tissue, particularly under specific physiological conditions like caloric restriction [233].

#### 4.3.2 The Immune–Metabolic Crosstalk in BAT

BAT hosts a dynamic immunometabolic microenvironment where immune cells reciprocally regulate thermogenesis and energy expenditure.

The adipose tissue macrophage (ATM) population is central to this dialogue. In obesity, ATMs expand from ∼5% to up to 50% of stromal cells and shift from a uniformly distributed state to forming “crown‐like structures” around dead adipocytes [234, 235]. These cells exhibit profound heterogeneity beyond the M1/M2 paradigm, defined by markers like CD11c, Ly6C, and CX3CR1 [236, 237]. Other innate immune cells, including neutrophils, dendritic cells, and mast cells, infiltrate and expand in obesity, while protective eosinophils decrease, a process regulated by Type 2 innate lymphocytes [238, 239, 240, 241, 242, 243].

Adaptive immunity also plays a key role. Obesity triggers an expansion of CD4^+^ and CD8^+^ T cells and a loss of anti‐inflammatory regulatory T cells in fat. The restricted T cell receptor repertoire suggests antigen‐driven responses, potentially to lipid antigens presented by adipocytes themselves, which can express MHC‐II under metabolic stress [244, 245, 246, 247, 248, 249, 250, 251].

Metabolic signals drive this immune recruitment. Lipolysis products function as endogenous danger signals, and tissue hypoxia in expanding fat depots activates HIF1α to promote inflammation. Conversely, upon cold exposure, M2 macrophages can produce catecholamines to stimulate thermogenesis in an IL‐4R‐dependent manner, highlighting the context‐dependent nature of the immune–metabolic crosstalk [252, 253].

#### 4.3.3 BAT in Multiorgan Communication Networks

**FIGURE 4:**
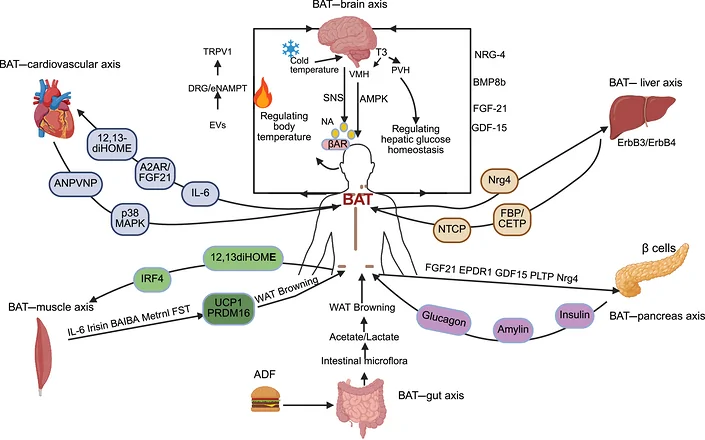
Systemic metabolic crosstalk mediated by BAT as an endocrine and signaling hub. Brown adipocytes generate heat via canonical UCP1‐dependent proton leakage, which uncouples oxidative phosphorylation. This is complemented by three ATP‐consuming futile cycles: creatine cycling, Ca^2^^+^ cycling via RyR/SERCA, and triglyceride–fatty acid cycling. These UCP1‐independent mechanisms provide alternative thermogenic routes, particularly relevant when UCP1 activity is limited or in beige adipocytes.

BAT is integrated into a sophisticated bidirectional communication network with multiple distal organs, including the brain, liver, skeletal muscle, cardiovascular system, gut, and pancreas, working in concert to maintain systemic energy homeostasis (Figure 4).

##### 4.3.3.1 The BAT–Brain axis

Serving as the central regulatory tier of this network, the BAT–brain axis is primarily governed by the hypothalamus. Hypothalamic outputs directly stimulate BAT thermogenesis via the SNS. The key neurotransmitter NE binds to β3‐adrenergic receptors on brown adipocytes, triggering the cAMP–PKA–HSL signaling cascade to initiate lipolysis and heat production [254]. This central regulation is multifaceted. Thyroid hormone T3 fine‐tunes the BAT thermogenic program via the hypothalamic AMPK pathway, while concurrently regulating hepatic glucose homeostasis through actions in the PVN, illustrating a neuro‐metabolic integrative mechanism [255]. Furthermore, estrogen modulates energy balance by reducing hypothalamic ceramide levels and endoplasmic reticulum stress. Leptin and insulin also converge on hypothalamic neurons to coordinately balance energy intake and expenditure by promoting BAT thermogenesis [256].

##### 4.3.3.2 The BAT–Liver Axis

A critical bidirectional dialogue is maintained between BAT and the liver. In addition to the established role of Nrg4 in suppressing hepatic lipogenesis, the liver reciprocally supports BAT thermogenesis by supplying acylcarnitine as metabolic substrates. This metabolic cooperation is further exemplified by liver‐derived bile acids, which activate the BAT BA–TGR5–cAMP–D2 signaling pathway to promote energy expenditure, and involves FGF21 in a bidirectional circuit that is pivotal for neonatal thermogenic activation [257, 258].

##### 4.3.3.3 The BAT–Muscle Axis

Communication between BAT and skeletal muscle is precisely coordinated through an exchange of myokines and batokines. Exercise induces skeletal muscle to secrete irisin, which promotes WAT browning and activates NST [259]. Concurrently, BAT releases 12,13‐diHOME, identified as a significant “exercise factor” that enhances fatty acid uptake and oxidation in skeletal muscle, thereby underpinning metabolic adaptation to exercise. This bidirectional crosstalk further involves other myokines such as β‐aminoisobutyric acid and METRNL, which collectively facilitate adipose tissue browning and thermogenesis through distinct signaling pathways [260].

##### 4.3.3.4 The BAT–Cardiovascular Axis

The BAT–cardiovascular axis demonstrates particularly important protective functions. BAT exerts pleiotropic cardioprotective effects by secreting factors including FGF21, NRG4, and 12,13‐diHOME. BAT‐derived FGF21, activated via an adenosine A2A receptor‐dependent mechanism, attenuates hypertension‐induced cardiac remodeling through the AMPK/PGC1α pathway [261]. NRG4, in turn, inhibits inflammatory responses and oxidative stress by activating the AMPK/NF‐κB and AMPK/NRF2 axes, thereby protecting against myocardial fibrosis and endothelial dysfunction [262]. Clinical evidence substantiates these findings, demonstrating a significant correlation between the presence of active BAT and a lower incidence of cardiometabolic diseases [263].

##### 4.3.3.5 The BAT–Gut Axis

The communication between BAT and the gut involves a complex gut–brain–BAT circuit. Gastrointestinal hormones such as GLP‐1 and glucagon enhance BAT thermogenesis by activating the SNS, mediating postprandial thermogenesis and satiety [264]. Gut microbiota and their metabolites, including butyrate and myristic acid, profoundly influence BAT function and WAT browning by modulating UCP1 expression and facilitating BAT development [265, 266].

##### 4.3.3.6 The BAT–Pancreas Axis

As a central metabolic regulator, the pancreas establishes functional reciprocity with BAT. BAT indirectly modulates pancreatic β‐cell function by enhancing systemic insulin sensitivity, while pancreatic hormones are postulated to provide feedback regulation of BAT activity, thereby closing a key metabolic feedback loop [267, 268, 269].

#### 4.3.4 Nontraditional Substrate Metabolism in BAT

Beyond canonical fuels, BAT oxidizes alternative substrates to support thermogenesis. Human BAT actively takes up glutamate during cold exposure, as confirmed by microdialysis studies [270]. Furthermore, cold‐induced systemic clearance of BCAAs is enhanced in individuals with active BAT, a process mediated by the mitochondrial transporter SLC25A44. Genetic evidence from mouse models confirms the functional importance of this pathway, as its disruption impairs thermogenesis [271]. In addition to amino acids, the TCA cycle intermediate succinate serves as a key thermogenic regulator. It accumulates in BAT upon cold exposure and directly stimulates thermogenesis, as demonstrated by supplementation studies in mice [271, 272].

## 5 BAT in Human Diseases, Aging, and Therapeutics

### 5.1 BAT in Pathophysiological States

#### 5.1.1 Obesity and Metabolic Disorders

In the context of obesity and metabolism, BAT counteracts energy accumulation primarily through thermogenesis mediated by UCP1, a process activated by cold exposure and diet‐induced thermogenesis [273]. The systemic regulation of energy expenditure involves compensatory mechanisms, as evidenced by mice that are deficient in UCP1 and exhibit enhanced thermogenesis in tissues such as skeletal muscle [274]. Following the challenges in clinically targeting BAT adrenergic pathways such as the ADRB3 receptor, the field has pivoted to explore its endocrine roles as a new frontier [275, 276]. BAT transplantation improves systemic glucose homeostasis via IL‐6 and FGF21‐dependent mechanisms, and its insulin signaling is crucial for sustaining pancreatic β‐cell function [277]. Furthermore, BAT contributes to lipid clearance by rapidly assimilating blood TGs upon cold activation. Clinically, the presence of active BAT in individuals with obesity is correlated with superior metabolic phenotypes, including enhanced insulin sensitivity and reduced visceral adiposity, underscoring its therapeutic potential even in a state of potentially diminished activity [278].

#### 5.1.2 Cancer and Cancer‐Associated Cachexia

BAT engages in a complex, bidirectional relationship with cancer that can be either antagonistic or facilitative [279, 280]. The tumor‐suppressive potential of BAT is mediated through its intense thermogenic activity, which creates a metabolic sink effect, competing for glucose and lipids and thereby imposing metabolic reprogramming and stress on cancer cells [281]. In a detrimental reversal, some cancers, like gastric carcinoma, actively promote BAT activation and WAT browning, which mechanically drive as a mechanism to drive the energy expropriation underlying cachexia [282]. Nevertheless, evidence also exists for a protective role of BAT in mitigating cachexia in other scenarios, highlighting the profound dependency of this interaction on the specific malignancy and the host's overall metabolic landscape [283].

#### 5.1.3 Reproductive Function and Fertility

BAT is increasingly recognized as a significant modulator within the metabolic‐reproductive axis, exerting protective effects on fertility in both sexes [284]. In female models of polycystic ovary syndrome (PCOS), whether induced pharmacologically by compounds like rutin or through cold exposure, the activation of BAT effectively alleviates ovarian dysfunction, leading to improved reproductive outcomes [285]. Furthermore, BAT transplantation can delay ovarian aging and extend reproductive lifespan in aged mice, potentially through the systemic modulation of IL‐6 and adiponectin [286]. In males, BAT transplantation has been demonstrated to counteract diet‐induced obesity's detrimental effects on sperm quality and fertility [287]. Mechanistically, BAT‐derived endocrine factors, including irisin and leptin, are known to directly stimulate steroidogenesis in human ovarian granulosa cells, providing a molecular basis for its direct regulation of reproductive endocrine function [288].

#### 5.1.4 Cardiovascular Diseases and Atherosclerosis

BAT confers significant protection against cardiovascular diseases, notably atherosclerosis. This benefit is primarily mediated through two parallel mechanisms, the systemic clearance of FFAs and the secretion of cardioprotective batokines such as adiponectin and FGF21 [289, 290]. Importantly, both of these beneficial processes can be augmented by cold exposure or pharmacological approaches that promote adipose tissue browning [291].

#### 5.1.5 Circadian Rhythm Disruption and Neuropsychiatric Risk

A complex interplay exists between BAT activity and the circadian system, with significant implications for metabolism and mental health [292]. The central and peripheral clocks tightly regulate BAT thermogenesis and the browning of WAT. Environmental challenges to circadian integrity, such as shift work or aberrant light exposure, disrupt this regulation, leading to BAT suppression and promoting metabolic dysfunction. The association between chronic long‐day photoperiods and increased fat accumulation underscores the sensitivity of this energy‐expending tissue to light cues [293]. This nexus among BAT, circadian rhythms, and photoperiod provides a plausible biological substrate for its indirect role in the etiology of mental illness. From a clinical perspective, the metabolic disturbances induced by certain antipsychotic drugs, such as clozapine, are mechanistically linked to their inhibitory effect on BAT energy expenditure [294].

#### 5.1.6 Systemic Inflammation and Immunometabolic Diseases

BAT functions as a significant immunomodulatory hub, actively mitigating systemic inflammation [295]. It fosters an anti‐inflammatory milieu by orchestrating a shift in cytokine profiles through upregulating anti‐inflammatory mediators like IL‐4 and IL‐10 while suppressing proinflammatory signals such as IL‐1β and IL‐6 [296]. Concurrently, it drives the polarization of ATMs toward the anti‐inflammatory M2 phenotype [297]. This coordinated immunomodulatory capacity is considered a principal mechanism through which BAT transplantation or activation confers systemic metabolic benefits, including enhanced glucose tolerance and insulin sensitivity.

### 5.2 The Waning Flame: Age‐Related Involution of BAT

The function and activity of BAT exhibit profound changes across the lifespan. Aging represents a primary determinant of BAT activity, characterized by a general decline in its quality and function, as well as alterations in its anatomical distribution [298]. This degenerative process is closely associated with systemic metabolic deterioration.

#### 5.2.1 Structural and Functional Decline of BAT with Age

BAT is most developed during infancy, where it is critical for neonatal thermoregulation. Its activity increases during puberty, a period synchronized with skeletal muscle development [299]. However, a progressive decline in both BAT mass and function commences in adulthood. In humans, active BAT depots are primarily located in the supraclavicular region, with secondary distributions in the mediastinum, paravertebral, epicardial, and abdominal areas [299]. With advancing age, superficial depots like interscapular regress first, while deeper perivascular and perirenal adipose tissues are lost later in life. ^18^F‐FDG PET‐CT studies demonstrate a marked age‐dependent reduction in cold‐induced BAT activity [300]. The detection rate of active BAT is threefold higher in individuals under 50 years compared with those over 64 years of age, with the decline potentially plateauing around the age of 60 years. This finding is consistent with the diminished cold tolerance and impaired thermoregulation observed in the elderly. In both rodent and human studies, aging BAT undergoes progressive infiltration by unilocular white adipocytes, a “whitening” process accompanied by a significant decline in UCP1 expression and thermogenic capacity [301].

#### 5.2.2 Molecular and Physiological Mechanisms Underlying Age‐Related BAT Decline

The deterioration of BAT with age is multifactorial. Mitochondrial integrity is central to BAT function, and aging is associated with accumulating mitochondrial DNA mutations, alongside diminished biogenesis and oxidative phosphorylation capacity [302]. Crucially, UCP1 activity and expression are significantly downregulated, directly compromising thermogenic efficiency. This is further compounded by an impaired regenerative potential of brown adipocyte progenitors [303].

SNS, the primary activator of BAT thermogenesis, exhibits reduced outflow to adipose tissue in older individuals. Concurrently, BAT responsiveness to NE is attenuated, partly due to a downregulation of β‐adrenergic receptors and catecholamine resistance induced by local inflammation [304].

Age‐related hormonal shifts also contribute to BAT decline. Sex hormones, such as estrogens and androgens, which positively correlate with BAT function, diminish in late adulthood, adversely affecting its activity [305]. T3, a key regulator of UCP1, decreases in serum concentration with age, coupled with reduced activity of Type II deiodinase, limiting local T3 bioavailability [306]. An imbalance in appetite‐regulating peptides emerges, with rising plasma ghrelin (a UCP1 inhibitor) against stable levels of obestatin (a UCP1 promoter), creating a net inhibitory environment for thermogenesis [307]. Furthermore, despite elevated circulating FGF21 levels in aging, adipose tissue often develops a state of “FGF21 resistance,” blunting its browning and thermogenic effects [308].

Aging adipose tissue typically enters a chronic low‐grade inflammatory state, termed “inflammaging”. This is characterized by increased infiltration of proinflammatory immune cells and mediators like TNF‐α. Such cytokines can directly suppress UCP1 and β3‐adrenergic receptor expression and indirectly impair BAT insulin sensitivity and overall function via activation of NF‐κB and MAPK signaling pathways [309].

The age‐related impairment in the browning of WAT involves several mechanisms. There is a reduction in the number and/or function of beige adipocyte progenitors expressing markers like CD137/TMEM26. Additionally, declining levels of the deacetylase SIRT1 during aging weaken its inhibitory effect on p53, thereby suppressing the differentiation of adipose‐derived mesenchymal stem cells into beige adipocytes [310]. The miRNA miR‐34a, a known negative regulator of SIRT1, plays a significant role in this regulatory node [311].

#### 5.2.3 Systemic Clinical Consequences of Age‐Related BAT Decline

The functional decline of BAT with age has far‐reaching health implications. A reduced thermogenic capacity lowers basal energy expenditure, potentially leading to a mild but persistent positive energy balance that contributes to mid‐life weight gain and obesity maintenance [312]. The loss of BAT‐mediated glucose disposal exacerbates systemic insulin resistance and hyperglycemia risk [313]. A diminished capacity to clear circulating lipids contributes to dyslipidemia. An inadequate thermogenic response directly underlies the poor cold tolerance and heightened susceptibility to hypothermia in the elderly [314]. Given that BAT presence is independently associated with a lower prevalence of cardiovascular conditions, its functional decline indirectly elevates cardiovascular risk by aggravating metabolic disorders and chronic inflammation, thereby negatively impacting the trajectory of healthy aging [315].

## 6 In Vivo Assessment of BAT: A Bridge From Biology to Function by Clinical Imaging Modalities

The mass of BAT refers to the actual weight of the tissue, independent of stimulation such as cold exposure or β‐agonist treatment. In contrast, the activation state of BAT, which can be reflected through imaging signals or functional measurements, is highly dependent on various factors including temperature, hormones, diet, medication, and SNS activity [316]. Given the interrelated nature of BAT mass and activation state under varying conditions [317, 318], accurately reporting BAT mass remains a significant challenge for imaging scientists. Critically, BAT does not exhibit an absolute “resting” state, and in living organisms, its activation level never reaches true zero. Consequently, distinguishing between BAT mass and activation state represents an exceptionally challenging endeavor.

**FIGURE 5:**
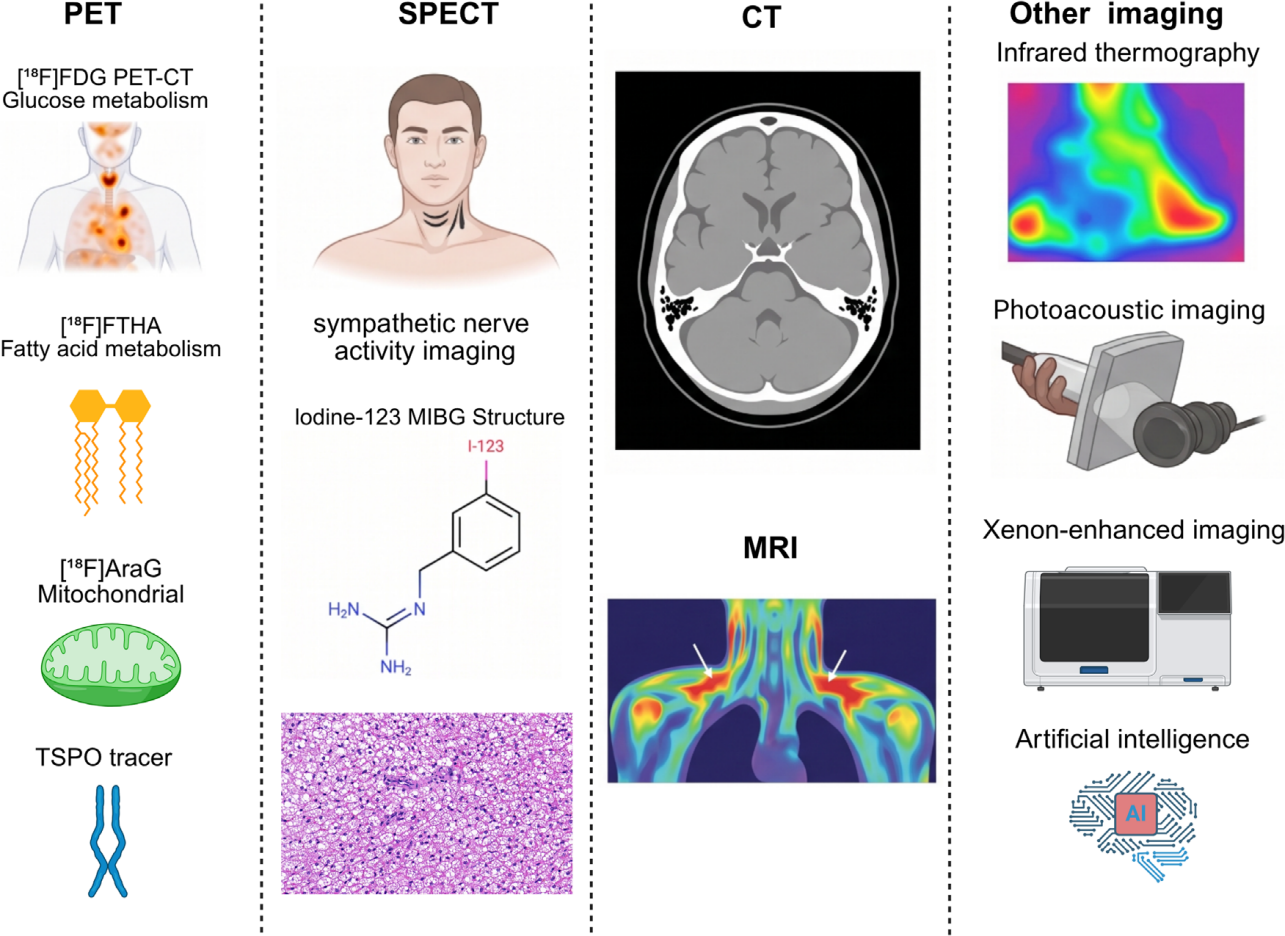
Multimodal imaging strategies for the in vivo assessment of BAT. BAT is assessed by multimodal imaging. PET (^1^ ^8^F‐FDG, ^1^ ^8^F‐FTHA) and SPECT (^1^ ^2^ ^3^I‐MIBG) measure metabolism and sympathetic activity; CT provides anatomical reference. MRI (fat‐fraction, perfusion) and infrared thermography offer radiation‐free functional assessment. Photoacoustic and xenon imaging add complementary readouts. AI‐based radiomics and deep learning further enhance automated BAT detection, quantification, and phenotyping.

The development of standardized, reproducible, and noninvasive imaging methods capable of assessing both BAT mass and activation would substantially advance the monitoring of therapeutic progress in BAT‐related research (Figure 5).

### 6.1 Metabolic Imaging: The Gold Standard and beyond

PET, especially when combined with CT (PET‐CT), has long been considered the gold standard for BAT detection. Its strength lies in the ability to noninvasively visualize specific metabolic processes. Based on BAT physiology, tracers targeting glucose, fatty acids, and mitochondria have been developed and widely applied.

#### 6.1.1 Glucose Metabolism Imaging

As glucose is a key substrate for BAT metabolism, [^18^F]FDG PET‐CT has become the reference method for in vivo BAT detection. Since seminal studies in 2009 confirmed its presence in adult humans, [^18^F]FDG has been extensively used to quantify BAT volume and activity, identify influencing factors, and assess activation strategies [319]. However, [^18^F]FDG reflects glucose metabolism rather than BAT mass and only images actively metabolizing tissue. Thus, imaging requires cold or pharmacological stimulation and is influenced by clinical variables such as BMI, age, sex, and blood glucose levels [320]. To improve comparability across studies, Chen et al. proposed the BARCIST 1.0 criteria in 2016, defining BAT on PET (lean SUV ≥ 1.2) and CT (attenuation between −190 and −10 Hounsfield units) and standardizing preparation and imaging protocols [321]. Nonetheless, the cost, complexity, radiation exposure, and its occasional discordance with BAT function in contexts such as insulin resistance collectively limit the utility of [^18^F]FDG for longitudinal and large‐scale studies [322, 323].

#### 6.1.2 Fatty Acid Metabolism Imaging

Fatty acids serve as the primary fuel for BAT thermogenesis and represent ideal imaging targets. Commonly used tracers include 14(R,S)‐[^18^F]fluoro‐6‐thia‐heptadecanoic acid ([^18^F]FTHA) and [^11^C]palmitate [324, 325]. Studies have shown that [^18^F]FTHA can detect cold‐induced increases in fatty acid uptake by BAT. Blondin et al. reported that fatty acid tracer uptake remained detectable in diabetic patients and may more accurately reflect activated BAT than [^18^F]FDG. Mouse studies further revealed that BAT preferentially oxidizes fatty acids during cold exposure, with a distinct uptake pattern compared with glucose. However, the clinical translation of fatty acid tracers is hampered by several limitations, including short physical half‐lives‐particularly the 20‐min half‐life of carbon‐11‐labeled palmitate‐relatively lower uptake rates compared with [^18^F]FDG, and nonspecific physiological accumulation in the liver and bowel. Novel agents such as [^18^F]monoacylglycerol lipase inhibitors, [^18^F]oleic acid analogs, and [^123/125^I]BMIPP are under development to improve specificity [326, 327, 328].

#### 6.1.3 Mitochondrial Imaging

Imaging mitochondria, the organelles responsible for thermogenesis, can provide unique functional and structural insights. A novel tracer, [^18^F]AraG, targets mitochondrial DNA synthesis. Owing to the high mitochondrial content in BAT, it robustly reflects tissue activation [329]. A unique advantage is its ability to simultaneously assess BAT activity and systemic immune function, offering a platform to study neuro–immune–adipose interactions [330, 331, 332, 333, 334, 335].

The translocator protein (TSPO) is highly expressed on the outer mitochondrial membrane [333]. Tracers such as [^18^F]FMPBR28, [^18^F]FEPPA, and [^18^F]FDPA can capture total BAT volume, including inactive depots, enabling identification of BAT and even beige fat under nonstimulated conditions [334, 335].

#### 6.1.4 Assessing Sympathetic Innervation and Perfusion by SPECT

Single‐photon emission computed tomography (SPECT) offers an alternative nuclear imaging approach that is generally more accessible and lower in cost than PET, though it suffers from inferior spatial resolution, sensitivity, and longer acquisition times [336, 337, 338, 339]. With a wide range of available tracers, SPECT can be used to assess sympathetic innervation and perfusion in BAT. For example, [^123^I]MIBG can map sympathetic nerve activity, while [^99m^Tc]MIBI can reveal increased blood flow [340, 341]. Notably, studies using [^123/125^I]BMIPP demonstrated differential substrate preferences between beige and classical BAT depots, highlighting metabolic heterogeneity within adipose tissue. Uptake of this tracer may also correlate more closely with metabolic rate than [^18^F]FDG [342].

#### 6.1.5 CT: Density‐Based Screening for BAT

Given its speed, low cost, and widespread availability, CT has become a practical tool for BAT screening in large populations. BAT attenuation (approximately −71.6 HU) is higher than that of WAT (approximately −104.4 HU), and active BAT appears denser due to lipid depletion and hyperemia. Ahmadi et al. proposed −87 HU as an optimal cutoff for identifying active BAT [343]. However, density alone is insufficient to reliably distinguish BAT from WAT or inflammatory lesions, and measurements can vary with scan parameters. Nevertheless, BAT evaluation using routine chest CT remains a promising first‐line screening strategy [344].

### 6.2 MRI: Nonionizing Structural and Functional Assessment

MRI provides a nonionizing, versatile platform for BAT assessment, making it well suited for repeated and long‐term monitoring [345]. The strength of this technique lies in its rich soft‐tissue contrast and multiparametric capabilities, which together permit a detailed and comprehensive characterization of target tissues.

#### 6.2.1 Dixon MRI and Fat‐Fraction Mapping

This technique separates water and fat signals to quantify fat fraction, distinguishing lipid‐poor BAT from WAT. Studies have shown that BAT fat fraction decreases upon cold exposure, reflecting lipid droplet consumption [344]. However, the method is susceptible to artifacts, and the overlap in fat fraction between BAT and WAT can limit diagnostic accuracy [344, 346].

#### 6.2.2 T2‐Weighted Imaging and Relaxation‐Based Contrast

BAT exhibits shorter T2 relaxation times than WAT, largely due to its iron‐rich mitochondrial membranes. Ouwerkerk et al. established a T2 cutoff of 76 ms to differentiate the two tissue types, with 85% sensitivity and 95% specificity [347]. Still, T2 values are influenced by changes in oxygenation, blood flow, and temperature during BAT activation, complicating quantitative interpretation [348, 349].

#### 6.2.3 Chemical Exchange Saturation Transfer MRI

Chemical exchange saturation transfer (CEST) is an emerging molecular MRI technique that detects endogenous metabolites without contrast agents. Cai et al. successfully quantified BAT mass and activity in mice using creatine‐based CEST. This method demonstrated higher sensitivity than [^18^F]FDG PET and detected BAT at thermoneutrality. Its noninvasive, radiation‐free nature holds great clinical promise, though performance in conditions such as UCP1 deficiency requires further validation [350, 351, 352].

#### 6.2.4 Blood Oxygen Level‐Dependent MRI

Blood oxygen level‐dependent (BOLD) fMRI noninvasively monitors BAT activation by detecting changes in tissue oxygenation and perfusion. Studies by Chen and Panagia et al. have demonstrated its feasibility and potential in revealing BAT–heart metabolic crosstalk [342].

### 6.3 Other Emerging Imaging Technologies

#### 6.3.1 Infrared Thermography

Infrared thermography (IRT) indirectly infers BAT thermogenesis by measuring skin temperature increases over the supraclavicular region after cold exposure [353]. It is completely noninvasive and radiation free [354]. However, it is restricted to superficial depots and influenced by environmental conditions. The relationship between IRT signals and UCP1 expression remains unclear [355].

#### 6.3.2 Photoacoustic Imaging

Photoacoustic imaging (PAI) combines optical contrast with ultrasound resolution, enabling assessment of BAT metabolism via hemoglobin oxygenation dynamics or lipid–water composition. It has been successfully applied in both murine and human studies and supports longitudinal imaging, though limited penetration depth (2–5 cm) restricts its use to superficial deposits [356, 357].

#### 6.3.3 Xenon‐Enhanced Imaging

The lipophilicity of xenon and high perfusion of BAT make it a unique contrast agent for CT and MRI. Xenon‐enhanced CT visualizes BAT distribution and perfusion [358], while xenon‐enhanced MRI enables absolute temperature mapping [359, 360]. However, high cost, technical complexity, and the need for specialized equipment limit its clinical adoption [361].

### 6.4 Artificial Intelligence and Quantitative Image Analysis in BAT Imaging

Artificial intelligence (AI), particularly radiomics and deep learning, is revolutionizing the assessment of BAT in medical images. These techniques enhance the extraction of quantitative information and automate analytical processes, offering new avenues for objective and high‐throughput BAT characterization.

Radiomics enables the extraction and analysis of subvisual features from medical images that are difficult to discern by the human eye. Studies by Nazeri and Li et al. have demonstrated the feasibility of using radiomic analysis to identify BAT on both PET and CT images, establishing preliminary detection models based on these features [362, 363].

Deep Learning, utilizing architectures such as convolutional neural networks, allows for the automated segmentation and detection of BAT depots without manual delineation, significantly improving analysis efficiency. For instance, Erdil et al. developed a deep learning model based on CT data to predict the corresponding [^18^F]FDG uptake in BAT [364]. Similarly, Cheng et al. and Bhanu Prakash et al. have applied deep learning for the automatic segmentation of BAT in rodent and human MRI studies [365, 366].

However, the development of robust AI models faces a significant challenge: the scarcity of large, high‐quality training datasets. A particular difficulty arises from the complex and variable factors that activate BAT in retrospective data. Inactive BAT depots are readily misclassified as negative in such datasets, introducing substantial uncertainty into the ground‐truth annotations used for training [367].

Future solutions to these limitations will likely involve combining large‐scale retrospective datasets with data from prospective, standardized interventional studies. Coupling such imaging data with histological validation will be crucial for improving the accuracy, reliability, and generalizability of AI models in BAT research.

## 7 Therapeutic Targeting of BAT: From Lifestyle to Regenerative Approaches

### 7.1 Nonpharmacological Lifestyle Interventions

#### 7.1.1 Cold Acclimation

Cold is the most potent BAT activator, primarily via sympathetic NE release and β‐adrenergic receptor activation of the PKA–UCP1 pathway. Additional mechanisms include TRPM8 sensitization and gut microbiota‐derived short‐chain fatty acids like butyrate [368]. Molecularly, cold‐induced PGC‐1α is vital for beige adipocyte recruitment [369], and PPARγ regulates mitochondrial biogenesis via PGC‐1α [370]. Cardiomyocyte‐released NPs after cold exposure enhance WAT PGC‐1α and UCP1 expression via p38 MAPK [371]. While BAT is detectable in only ∼9% of adults under thermoneutral conditions [372], controlled cold exposure (16–19°C) for several hours daily over weeks increases BAT mass/activity, enhances cold‐induced thermogenesis, and reduces fat mass [373]. Winter swimmers exhibit stronger thermogenesis, and acute cold exposure reduces shivering while upregulating NST [374, 375], suggesting potential for restoring BAT activity in obesity. Challenges include discomfort and potential compensatory food intake [376, 377]. Mild cold exposure (20–22°C) also enhances BAT thermogenesis [378], and local hyperthermia activates BAT via HSF1 independently of NE. Additionally, exercise represents an alternative, inducing WAT browning via myokines independently of sympathetic stimulation [379].

#### 7.1.2 Exercise and Nutritional Modulation

Exercise beneficially remodels adipose tissue by reducing WAT volume, improving glucose tolerance, and enhancing mitochondrial activity [380]. In rodents, both aerobic and strength training promote WAT browning via myokines, such as irisin, FGF21, and sympathetic activation [381, 382]. However, human evidence is less consistent that some studies note enhanced thermogenic gene expression in subcutaneous fat, the ACTIBATE trial found no change in BAT volume or activity after 24 weeks of combined training [383]. Endurance athletes exhibit lower BAT activity than sedentary individuals, suggesting skeletal muscle may compensate for BAT thermogenesis [384]. Meanwhile, dietary interventions show potential that intermittent fasting shapes gut microbiota and promotes beige adipose thermogenesis, and postprandial insulin stimulates BAT glucose uptake [385]. Specific nutrients, including n‐3 PUFAs, increase UCP1 and PRDM16 in human subcutaneous adipocytes, though direct BAT activation is unconfirmed [386]. Green tea catechins enhance energy expenditure by inhibiting catecholamine degradation [387], and phytochemicals such as curcumin, resveratrol promote browning in animals via AMPK/SIRT1/PGC1α [388], but human evidence is limited by bioavailability.

### 7.2 Pharmacological Activation by Direct Activators

#### 7.2.1 Sympathomimetic and β‐Adrenergic Agents

Clinical outcomes for agents mimicking catecholamines to activate BAT β‐adrenergic receptors are complex. While acute ephedrine administration variably affects BAT glucose uptake in healthy individuals, chronic use reduces fat mass without altering resting energy expenditure and may diminish BAT ^1^ ^8^F‐FDG uptake, indicating potential tolerance [389, 390]. Historical agents like phentermine and sibutramine induce moderate weight loss but are limited by rapid tolerance and cardiovascular risks [391]. Administration of the β3‐adrenergic receptor agonist mirabegron at a 200 mg dose effectively activates human BAT and significantly increases systemic energy expenditure. This demonstrates the feasibility of pharmacologically targeting human BAT and provides proof‐of‐concept for a novel therapeutic strategy against obesity and related metabolic diseases [392]. Based on the study, long‐term administration of the β3‐adrenergic receptor agonist mirabegron (100 mg/day for 4 weeks) was shown to directly enhance metabolic activity in human BAT and increase resting energy expenditure. Additionally, the treatment conferred broad metabolic benefits, including improved insulin sensitivity, enhanced β‐cell function, elevated beneficial lipoprotein levels such as HDL, and increased adiponectin. These effects demonstrate that activating β3‐AR is a feasible strategy for treating metabolic diseases. However, the dose used in the study (100 mg) also induced increases in heart rate and blood pressure, indicating that cardiovascular safety during long‐term application still requires careful attention [393].

#### 7.2.2 PPARγ Agonists

Despite inducing WAT browning in vitro and in rodents, human evidence for PPARγ agonists like pioglitazone is inconsistent [394]. One study reported reduced cold‐induced BAT glucose uptake after 28 days of treatment, while another observed increased WAT browning markers without enhanced BAT activity after 12 weeks (Table 2). The contribution of WAT browning to their antidiabetic effects in humans remains unclear, and these drugs are unlikely to activate human BAT metabolism [395].

**TABLE 2:** Effects of drug intervention on metabolism of human brown adipose tissue.

Reg. #	Design	Results	References
NCT02236962 (ephedrine)	Recruited 23 healthy young men who were either given ephedrine (1.5 mg/kg/day) or a placebo of lactose for 28 days.	Long‐term use of ephedrine inhibits glucose utilization of BAT, indicating that long‐term use of ephedrine reduces rather than increases BAT activity.	[389]
NCT01015794 (ephedrine)	Nine young men of slender build and nine obese men each received 2.5 mg/kg orally of ephedrine, a highly bioavailable sympathomimetic drug; and received the same placebo on another day.	Compared with placebo, ephedrine increased BAT activity in lean subjects but had no effect on obese subjects. Changes in BAT activity with ephedrine, compared with placebo, were negatively correlated with several body fat percentages.	[390]
NCT01783470 (Mirabegron)	Twelve subjects were given a single oral dose of 200 mg mirabezon or a placebo.	β3‐adrenergic receptor agonists can stimulate thermogenesis in human brown adipose tissue.	[392]
NCT03049462 (mirabegron)	Fourteen healthy women were enrolled and received mirabezon (Myrbetriq extended‐release tablets, Astellas Pharma) for 4 weeks at a dose of 100 mg.	Long‐term mirabezonitrogen treatment can enhance the metabolic activity of BAT. Intravenous glucose tolerance tests showed improvements in insulin sensitivity, glucose utilization, and insulin secretion.	[393]
NCT02596776NCT02919176(mirabegron)	Study participants who were underweight or obese received either cryotherapy (applying ice packs to the upper thighs for 30 min daily for 10 days) or the β3 receptor agonist mirabezon.	Long‐term (10 weeks; 50 mg/day) treatment with the β3 receptor agonist mirabezon induced the expression of UCP1, TMEM26, and CIDEA, as well as the phosphorylation of HSL at serine 660 in obese subjects.	[394]
NCT02236962 (pioglitazone)	Male subjects without cardiovascular metabolic disease (lactose; *n* = 7, age 22 ± 1 year) or pioglitazone (45 mg/day; *n* = 7, age 21 ± 1 year) for 28 days.	Pioglitazone significantly promoted browning and adipogenesis in adipocytes in vitro.	[396]
NCT02919176 (pioglitazone)	Mirabelon (50 mg), pioglitazone (30 mg), or a combination of both, were administered once daily for 12 weeks. All medications were purchased from the hospital pharmacy. This was an open‐label study.	Treatment with pioglitazone or pioglitazone in combination with mirabezon can increase the expression of beige adipose tissue protein markers and improve insulin sensitivity and glucose homeostasis.	[397]
NCT02964442 (capsaicin)	Twenty healthy adult men and women participated. The study used 12 mg of capsaicin and a 14.5°C cooling vest as interventions.resveratrol) improved 6‐min walk distance compared with placebo, with assessments conducted at 6 months of follow‐up.	After ingesting capsaicin‐like substances, the energy expenditure (EE) of participants with positive brown adipose tissue (BAT) increased by approximately 10% compared with those with negative BAT (approximately 5%).	[398]

#### 7.2.3 Other Receptor Agonists and Natural Compounds

TRPV1 agonists like capsaicin promote weight loss via satiety and catecholamine release [399]. Chronic supplementation in humans increases resting metabolic rate, cold‐induced thermogenesis, and supraclavicular ^18^F‐FDG uptake, suggesting enhanced vascularization, yet acute administration does not augment BAT glucose uptake, and direct thermogenic evidence is lacking [396]. The TRPM8 agonist menthol increases UCP1 expression and prevents obesity in rodents [397].

While topical L‐menthol has been associated with modestly elevated energy expenditure in humans, direct proof of BAT activation remains elusive. More compelling preliminary evidence comes from TGR5 agonists like chenodeoxycholic acid. A small human study reported that such treatment increased both BAT ^18^F‐FDG uptake and basal energy expenditure, a finding corroborated by in vitro experiments showing enhanced uncoupled respiration in human brown adipocytes [400]. These combined clinical and mechanistic insights offer a promising preliminary proof of concept. Green tea extracts, such as catechins and caffeine, augment energy expenditure, particularly in BAT‐positive individuals, by inhibiting catechol‐O‐methyltransferase and phosphodiesterase, though direct BAT thermogenic effects require confirmation. Although fish oil (DHA/EPA) promotes WAT browning in some models, evidence for human BAT activation is absent, and its antiobesity effects may be UCP1‐independent [398, 401, 402] (Table 2 summarizes the key clinical evidence on human BAT activation and metabolism by these agents).

### 7.3 Pharmacological Strategies by Metabolic Sensitizers and Enhancers

#### 7.3.1 Targeting SIRT1 Activation

SIRT1, an NAD^+^‐dependent deacetylase, is a central regulator of adipose tissue browning and thermogenesis. It promotes a thermogenic phenotype by deacetylating PPARγ, which recruits PRDM16 and suppresses the corepressor NCoR to initiate browning [403]. SIRT1 also activates PGC‐1α, driving UCP1 expression and mitochondrial biogenesis, and forms a positive feedback loop with AMPK to amplify energy metabolism signals [404, 405]. Preclinical studies confirm that activating the AMPK/SIRT1/PGC1α axis induces WAT thermogenesis, and SIRT1 synergizes with β3‐adrenergic receptor activation to stimulate lipid mobilization and thermogenesis. TRPV1‐mediated Ca^2+^ influx activates CaMKII/AMPK, leading to SIRT1‐dependent deacetylation of PPARγ and PRDM16 [406]. Conversely, SIRT1 deficiency in BAT impairs mitochondrial biogenesis and thermogenic gene expression [407]. SIRT1 also acts in skeletal muscle, deacetylating PGC‐1α to enhance FNDC5 cleavage and irisin release, which stimulates WAT browning systemically [408, 409].

#### 7.3.2 Targeting NAD^+^ Metabolism

**FIGURE 6:**
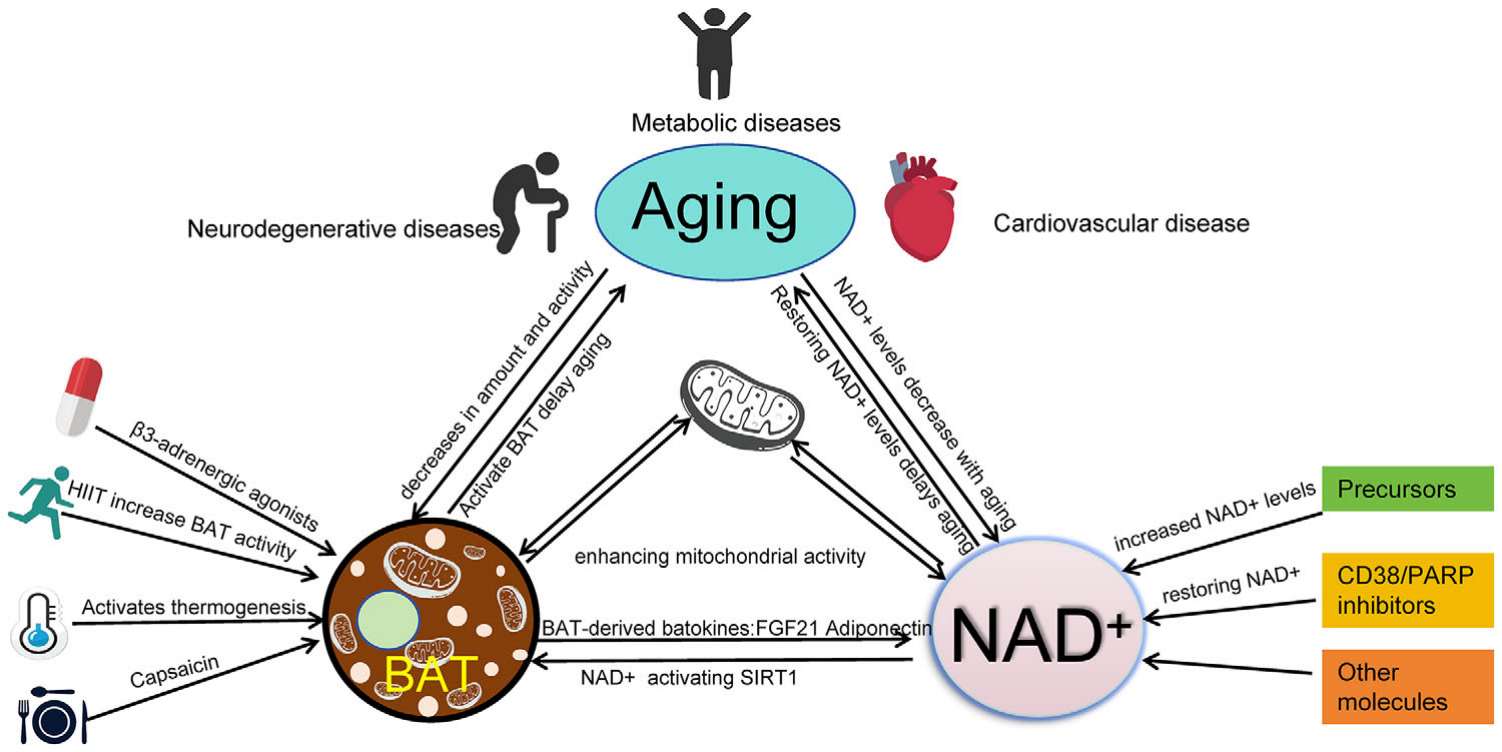
The interplay between aging, NAD^+^ metabolism, and BAT homeostasis. Aging reduces NAD^+^ levels, impairing sirtuin activity and mitochondrial function, leading to BAT whitening and metabolic decline. Therapeutic interventions target two fronts: enhancing BAT recruitment (cold, exercise, capsaicin, β3‐agonists) and restoring NAD^+^ pools (NR/NMN, CD38/PARP inhibition). Combined strategies addressing both pathways may counteract aging‐related metabolic dysfunction.

Intracellular NAD^+^ levels are crucial for mitochondrial function. NAD^+^ depletion is linked to impaired BAT function in metabolic diseases, while elevating NAD^+^ promotes mitochondrial activity and thermogenesis [410, 411]. Exogenous NAD^+^ enhances NE‐stimulated uncoupling in human brown adipocytes [412]. The rate‐limiting enzyme in NAD^+^ biosynthesis, NAMPT, is essential for adaptive thermogenesis; adipose‐specific NAMPT knockout mice exhibit cold intolerance and BAT “whitening,” reversible by NMN supplementation [413]. Another precursor, NR, also induces murine thermogenesis with increased UCP1 and PGC‐1α [414]. Mitochondrial‐associated AIFM2, upregulated by cold or β‐adrenergic stimulation, supports glycolysis for thermogenesis by regenerating cytoplasmic NAD^+^ and its knockout impairs thermogenesis and increases adiposity [415]. Inhibiting NAD^+^‐consuming enzymes like PARP1 and CD38, which compete with sirtuins for NAD^+^, enhances BAT function. PARP1 knockout increases BAT NAD^+^ levels, SIRT1 activity, and mitochondrial biogenesis [416]. CD38 is upregulated in obesity, and its knockout protects against diet‐induced obesity via the SIRT1/PGC1α axis [417]. Cold exposure downregulates CD38 of BAT and finally increase NAD^+^[418]. Thus, elevating NAD^+^ through precursors, biosynthetic enhancers, or consumer inhibition represents a promising strategy to potentiate BAT thermogenesis (Figure 6).

### 7.4 Cell‐Based Therapy: BAT Transplantation and Adipokine Restoration

BAT transplantation improves metabolic phenotypes in animal models primarily by activating endogenous BAT rather than via the graft's own thermogenesis [419]. It acts through an adipokine network. Briefly, BAT‐derived IL‐6 is essential for improving insulin sensitivity, and transplantation increases adiponectin and enhances β‐adrenergic signaling and fatty acid oxidation in WAT [420]. In Type 1 diabetic models, embryonic BAT transplantation reverses symptoms independently of insulin, correlating with increased IGF‐1, and establishes a new metabolic equilibrium in nonobese diabetic mice [421]. Benefits extend to improving PCOS symptoms and conferring resistance to diet‐induced obesity via enhanced sympathetic tone [422, 423]. Transplantation enhances glucose uptake in endogenous BAT, WAT, and myocardium but not skeletal muscle [424]. Translational challenges include significant interspecies differences in batokine profiles [343].

### 7.5 Translational Hurdles, Species Differences, and Future Perspectives

Translating BAT research from rodents to humans is challenged by profound interspecies differences. Anatomically, rodent BAT is primarily interscapular and β3‐adrenergically dominated, whereas human BAT is diffusely distributed and primarily β2‐adrenergically regulated, explaining the failure of many rodent‐effective β3 agonists in clinical trials. Physiologically, human BAT activity declines with age and obesity, complicating detection and intervention. Methodologically, standard rodent housing (∼22°C) imposes chronic mild cold stress, potentially overstating BAT's metabolic role, whereas humans largely buffer environmental temperature. Clinical ^18^FDG‐PET/CT assesses glucose uptake but not direct thermogenic capacity, limiting functional assessment. Furthermore, intervention outcomes also diverge. Cold exposure activates human BAT but with limited weight loss due to compensatory appetite, and exercise promotes browning in rodents but is associated with lower BAT activity in human athletes, indicating possible tissue‐level metabolic competition. To address these species‐specific disparities, a multipronged approach is required. This entails pursuing targeted drug design for humans, developing physiological models at thermoneutrality, advancing multimodal imaging techniques for BAT, and ultimately exploring combinatorial therapeutic strategies. Acknowledging these differences is crucial for developing effective and safe BAT‐targeted therapies for humans.

## 8 Conclusion

Research on BAT has evolved from a narrow focus on thermogenesis to a broader appreciation of its role as a key regulator of systemic metabolic homeostasis. Beyond its energy‐expending function, BAT is now recognized as an active endocrine organ that significantly influences glucose and lipid metabolism. This paradigm shift has spurred the investigation of BAT activation and transplantation as promising therapeutic strategies for metabolic diseases.

The clinical translation of these findings, however, faces substantial hurdles. The current gold standard for BAT detection, [^18^F]FDG PET‐CT, is limited by its protracted procedure and radiation exposure, restricting its widespread use. While emerging techniques like magnetic resonance and PAI offer potential for noninvasive, longitudinal monitoring, they currently lack standardized diagnostic criteria and robust validation. A fundamental challenge remains the difficulty in definitively distinguishing classical BAT from inducible beige adipocytes in humans, as these cell types share anatomical niches but differ in developmental origin and molecular regulation.

In the realm of therapeutics, studies on agents like the β3‐adrenergic receptor agonist mirabegron have been instructive. While demonstrating a capacity to increase energy expenditure and improve insulin sensitivity, even in individuals with low BAT mass, its clinical utility is counterbalanced by cardiovascular side effects, including tachycardia and elevated blood pressure. Furthermore, emerging evidence from animal models suggests that potent BAT activation might paradoxically exacerbate atherosclerosis through enhanced lipolysis, underscoring the critical need to carefully balance efficacy with long‐term safety in therapeutic development.

Future research should prioritize several key directions. First, the establishment of precise, noninvasive, and standardized methodologies for quantifying human BAT mass and activity is paramount. Second, a deeper exploration of the BAT secretome and the physiological roles of specific “batokines” will unveil novel endocrine targets beyond thermogenesis. Finally, elucidating the role of epigenetic regulators, such as long noncoding RNAs, in controlling the adipocyte thermogenic program holds promise for uncovering fundamentally new therapeutic avenues.

In summary, BAT research stands at a pivotal transition from basic discovery to clinical application. By integrating insights from molecular biology with advanced imaging technologies, and by refining both existing and novel intervention strategies, the full therapeutic potential of targeting this dynamic tissue can be realized, offering new avenues for combating metabolic diseases.

## Author Contributions

C. Sun and L. Lu designed this review study, while X. Deng, Y. Shen, and Y. Sun collected the materials for the manuscript. X. Deng primarily wrote the manuscript and prepared the figures and tables, and C. Sun edited it. All authors have read and approved the final manuscript.

## Ethics Statement

The authors have nothing to report.

## Conflicts of Interest

The authors declare no conflicts of interest.

## Data Availability

The authors have nothing to report.
